# A Universal Strategy to Enhance Polarization Performance and Anode Reversal Tolerance by Polyaniline‐Coated Carbon Support for Proton Exchange Membrane Fuel Cells

**DOI:** 10.1002/advs.202407570

**Published:** 2024-10-01

**Authors:** Zheng Li, Yongbiao Mu, Qing Zhang, Cailin Xiao, Yuting Jiang, Lei Du, Siyu Ye, Tianshou Zhao, Lin Zeng

**Affiliations:** ^1^ Department of Mechanical and Aerospace Engineering The Hong Kong University of Science and Technology Clear Water Bay Kowloon Hong Kong 999077 China; ^2^ Shenzhen Key Laboratory of Advanced Energy Storage Department of Mechanical and Energy Engineering Southern University of Science and Technology Shenzhen 518055 China; ^3^ SUSTech Energy Institute for Carbon Neutrality Southern University of Science and Technology Shenzhen 518055 China; ^4^ Huangpu Hydrogen Energy Innovation Centre School of Chemistry and Chemical Engineering Guangzhou University Guangzhou Guangdong 510006 China; ^5^ SinoHykey Technology Company Ltd. Guangzhou Guangdong 510760 China

**Keywords:** Anode catalyst layer, Anode reversal, Durability, Polyaniline‐coating carbon support, Proton exchange membrane fuel cell

## Abstract

Anode cell reversal typically leads to severe carbon corrosion and catalyst layer collapse, which significantly compromises the durability of proton exchange membrane fuel cells. Herein, three types of commercial carbon supports with various structures are facilely coated by polyaniline (PANI) and subsequently fabricated into reversal‐tolerant anodes (RTAs). Consequently, the optimized PANI‐coated catalyst RTAs demonstrate enhanced polarization performance and improved reversal tolerance compared to their uncoated counterparts, thus confirming the universality of this coating strategy. Essentially, the surface engineering introduced by PANI coating incorporates abundant N‐groups and enhances coulombic interactions with ionomer side chains, which in turn reduces lower carbon exposure, promotes more uniform Pt deposition, and ensures better ionomer distribution. Accordingly, the membrane‐electrode‐assembly containing the Pt/PANI/XC‐72R‐1+IrO_2_ RTA presents a 100 mV (at 2500 mA cm^−2^) polarization performance improvement and 26‐fold reduction in the degradation rate compared to the uncoated counterpart. This work provides a universal strategy for developing durable anodes and lays the groundwork for the practical fabrication of high‐performance, low‐degradation RTA.

## Graphical abstract

A universal PANI coating carbon support strategy is developed to decrease carbon exposure and facilitate uniform Pt deposition and ionomer distribution, thus improving polarization performance and anode reversal tolerance for PEMFC. The thickness of PANI coating should be optimized since the over‐thick coating will result in mass transfer problems.

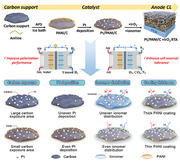

## 1 Introduction

Facing the dilemma of increasing global energy demands and carbon‐neutral concerns, hydrogen energy has been attracting more attention than ever due to its high energy density, abundant resources, and zero pollutant emissions.^[^ ^1^ ^]^ Among various hydrogen utilization devices, proton exchange membrane fuel cells (PEMFCs) stand out as the most advanced toward commercial viability, recognized as a powerful tool in driving the transition towards a carbon‐neutral society.^[^ ^2^ ^]^ However, cost and durability issues retard the PEMFCs from being broadly commercialized.^[^ ^3^, ^4^, ^5^, ^6^ ^]^ Specifically, the inevitable anode reversal, which can be caused by fuel starvation, uneven gas distribution, startup/shutdown operations, and/or rapid load variations, can lead to high potential in the anode, posing a substantial risk to the durability of PEMFCs in terms of carbon support corrosion and catalyst layers collapse.^[^ ^7^ ^]^ Once anode reversal takes place, the regular hydrogen oxidation reaction (HOR) is suppressed, allowing the harmful carbon corrosion reaction (COR) competes with the oxygen evolution reaction (OER) in providing sufficient electrons and proton flows.^[^ ^8^ ^]^

(1) $$2H_{2}O\to H^{+}+O_{2}+4e^{-}\;\;E^{0}=\;1.23V\;\left(\mathrm{vs}.\mathrm{RHE}\right)$$

(2) $$C+2H_{2}O\to CO_{2}+4H^{+}+4e^{-}\;\;E^{0}=\;0.21V\;\left(\mathrm{vs}.\mathrm{RHE}\right)$$

(3) $$C+H_{2}O\to \mathrm{CO}+2H^{+}+2e^{-}\;\;E^{0}=\;0.52V\;\left(\mathrm{vs}.\mathrm{RHE}\right)$$

To avoid anode reversal, tailored control strategies for fuel cell systems are employed to prevent fuel starvation. Nevertheless, the inherent hysteresis in such control systems makes it difficult because of the uneven distribution of substantial active regions within individual commercial cells and the complex fuel diffusion pathways within porous electrodes.^[^ ^9^ ^]^ The inevitability of anode reversal requires a more fundamental material innovation. Traditionally, most studies focused on developing novel OER catalysts to boost OER kinetics and thus minimize the destruction of COR.^[^ ^10^, ^11^, ^12^ ^]^ However, polarization performance degradation has been reported to persist during the OER‐dominant period, indicating that carbon corrosion cannot be eliminated.^[^ ^13^ ^]^ Therefore, enhancing the inherent corrosion resistance of carbon supports during anode reversal is imperative, and, importantly, without sacrificing the polarization performance. Although using high‐degree graphitized carbon is beneficial in alleviating the reversal damage, polarization performance is negatively impacted.^[^ ^14^, ^15^ ^]^ Hence, it is urgent to seek another novel strategy to protect the carbon support. Previous research of our group pointed out that decreasing the carbon exposure area or increasing the metal coverage effectively improves anode reversal tolerance.^[^ ^16^ ^]^ Although it can be achieved by applying higher metal content or lower specific surface area carbon support, it is noteworthy that this strategy always causes a super‐thin anode catalyst layer and potential Pt aggregation.^[^ ^17^ ^]^ Moreover, achieving low carbon exposure is challenging when using high specific surface area carbon, such as Ketjenblack, the one commonly applied in the fuel cell industry. Polyaniline (PANI) has been widely used in electrochemical energy storage and conversion technologies due to its high conductivity, low cost, environmental‐friendly properties.^[^ ^18^ ^]^ It can be synthesized by an in situ electrochemical polymerization method with the potential range controlled up to 1.1 V versus saturated calomel electrode (SCE).^[^ ^19^, ^20^ ^]^ It should be noted that the applying potential is limited by the water electrolysis potential (1.23 V) instead of the decomposition of polyaniline, which implies that the conductive‐polymer polyaniline may tolerate higher potential and fulfill the requirements for stable performance during cell reversal while maintaining high conductivity. Additionally, the in situ polymerization method allows for easy deposition on various surfaces and precise control of the deposition thicknesses, making the manipulation of PANI coating possible.

In this work, polyaniline‐coated carbon support (PANI/C) were facilely synthesized based on three carbon supports with different specific surface areas. PANI/C was subsequently employed as a support to prepare Pt/PANI/C catalysts. The fabricated reversal‐tolerant anodes (RTAs) based on Pt/PANI/C exhibited not only enhanced polarization performance but also improved reversal tolerance, compared with those using uncoated counterparts. Moreover, the thickness of PANI coating layers was optimized because it is evidenced that the excessively thick PANI coating causes side effects. The PANI‐coating layer protects the carbon support from the outer high potential and corrosion environment during the cell reversal, leading to an extended reversal time and decelerated degradation rate in polarization performance. Density functional theory (DFT) calculations simulated the thermodynamic structure stability of Pt/PANI/C under high potential, which explains the corrosion resistance during anode reversal. Further investigations verified by transmission electron microscope (TEM), energy dispersive spectrometer (EDS) mapping, and isothermal titration calorimetry (ITC) characterizations prove that the enhanced polarization performance is attributed to more uniform Pt deposition and even ionomer distribution, facilitated by the abundant N‐groups acting as nucleating centers and their coulombic interaction with negatively charged ionomer side chains.

## 2 Results and Discussion

**Figure 1:**
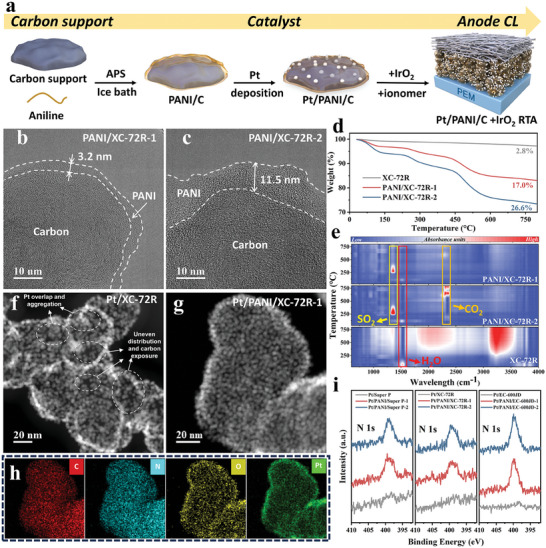
a) Schematic diagram of the synthesis procedure of Pt/PANI/C+IrO_2_ RTA. b,c) TEM images of PANI/XC‐72R with different PANI‐coating thickness. d,e) TG‐IR results for XC‐72R, PANI/XC‐72R‐1, PANI/XC‐72R‐2. f–h) TEM images of Pt/XC‐72R and Pt/PANI/XC‐72R‐1 and the corresponding EDS mapping. i) XPS N 1s spectra of all Pt/C and Pt/PANI/C samples.

The procedures of synthesizing Pt/PANI/C catalysts and the corresponding Pt/PANI/C+IrO_2_ RTAs are illustrated in **Figure** **1**a. The specific surface areas of the three carbon supports used in this study are Super P < XC‐72R < EC‐600JD, with values of 73, 218, and 1327 m^2^ g^−1^, respectively, as shown in Figure S1 (Supporting Information). TEM was used to investigate the thickness of PANI‐coating in PANI/C. As shown in Figure 1b,c, for XC‐72R carbon support, the PANI thickness derived from recipe 1 (carbon:aniline = 1.4:1) is ≈3.2 nm (this support is denoted as PANI/XC‐72R‐1), while the thickness of recipe 2 (carbon:aniline = 1.4:2) increases to 11.5 nm (this support is denoted as PANI/XC‐72R‐2), displaying a non‐linear relationship with different aniline contents. To confirm the PANI coating and investigate the mass fraction of PANI, the thermogravimetry‐infrared (TG‐IR) experiments were performed for XC‐72R, PANI/XC‐72R‐1, and PANI/XC‐72R‐2. The thermogravimetry (TG) result shown in Figure 1d indicates only 2.8% weight loss for XC‐72R until 800 °C, while 17.0% and 26.6% weight loss for PANI/XC‐72R‐1 and PANI/XC‐72R‐2, respectively, were obtained. The weight loss differences between XC‐72R and PANI‐coated XC‐72R indicate the successful coating of PANI on the carbon support surface, and the PANI/XC‐72R‐2 had more significant weight loss than PANI/XC‐72R‐1 due to its thicker PANI coating, as shown in Figure 1b,c. Meanwhile, the gas emission during the TG was detected by real‐time IR, as shown in Figure 1e. The PANI coating samples undergo three‐step thermal degradation processes. The first degradation step during 30–150 °C is attributed to the expulsion of residual moisture present in the PANI, confirmed by the IR signal at around 1600 cm^−1^ assigning to the O–H bending vibration of water. The second weight loss occurs in the temperature range of 220–300 °C, which is due to the loss of dopant H_2_SO_4_ from the PANI synthesis procedure, corresponding to the IR signal of SO_2_ at about 1335 cm^−1^.^[^ ^21^ ^]^ The third weight loss started at around 450 °C should be attributed to the degradation and decomposition of the backbone units of PANI as the XC‐72R support is thermally stable^[^ ^22^ ^]^ and the IR signal of CO_2_ at about 2358 cm^−1^ was observed.^[^ ^23^ ^]^ The difference in IR spectra between XC‐72R and PANI‐coating XC‐72R again confirms the successful coating of PANI. An identical TG test was performed on Super P and EC‐600JD yielding similar results, as shown in Figure S2 (Supporting Information).

X‐ray diffraction (XRD) patterns of Pt/PANI/C shown in Figure S3 (Supporting Information) confirm the successful deposition of Pt on the supports. Using the Scherrer equation, a similar Pt nanoparticle size (i.e., 3–4 nm) was estimated for all samples based on the Pt (220) peak. No PANI‐related peak is observed for Pt/PANI/C samples since the PANI is amorphous. The morphology of all homemade Pt/C and Pt/PANI/C was observed by transmission electron microscopy (TEM) and is shown in Figure S4 (Supporting Information). Specifically, Pt/XC‐72R and Pt/PANI/XC‐72R‐1 are presented in Figure 1f,g to highlight the Pt deposition difference with or without PANI‐coating under the same synthesis method and condition. It can be found that the Pt nanoparticles aggregate and overlap apparently when the Pt is directly deposited on the carbon surface without PANI coating, leading to increased carbon exposure. In contrast, the Pt/PANI/XC‐72R‐1 displays a uniform Pt distribution, and the carbon support is coated with a tightly connected Pt nanoparticle network. A similar phenomenon was observed for Pt/Super P and Pt/PANI/Super P, as shown in Figure S5 (Supporting Information), with more uniform Pt nanoparticle distribution for Pt/PANI/Super P‐1 than Pt/Super P. The EDS mapping of Pt/PANI/XC‐72R‐1 was also performed and the result is shown in Figure 1h. Pt element is uniformly and homogeneously distributed across the surface with abundant C and N elements, proving the presence of PANI after the Pt deposition. Similar EDS mapping was conducted for Pt/PANI/EC‐600JD‐1 and Pt/PANI/EC‐600JD‐2 to confirm the presence of the N element, as shown in Figures S6 and S7 (Supporting Information). The promoting effect of the PANI coating on Pt nanoparticle deposition primarily arises from the creation of a uniform carbon support environment with an even distribution of N elements. Previous reports have indicated that the introduction of the N element on the catalyst support facilitates the uniform distribution of Pt nanoparticles.^[^ ^24^, ^25^, ^26^ ^]^ The coating of PANI can not only mitigates the differences of the original carbon surface but also provides N‐groups working as nucleation centers, together promoting the even deposition of Pt nanoparticles. X‐ray photoelectron spectroscopy (XPS) was also performed to verify the presence of PANI in all samples after Pt deposition, as PANI is the sole source of N element in the Pt/PANI/C catalysts. Additionally, the content and distribution of N element provide significant insights into the coating properties of PANI. As shown in Figure 1i, no N 1s peak is observed for Pt/Super P, Pt/XC‐72R, and Pt/EC‐600JD, while the N 1s characteristic peak at around 400 eV appears for all PANI‐coated samples, further confirming the retention of PANI coating layers in the Pt/PANI/C catalysts. Moreover, it can be inferred that a thicker PANI coating layer results in a higher N element content. As shown in Table S1 (Supporting Information), the N 1s mass contents for Pt/PANI/C‐2 obtained by XPS are higher than those for Pt/PANI/C‐1 (≈2 wt.% for Pt/PANI/C‐1 and 3 wt.% for Pt/PANI/C‐2), which is consistent with the PANI coating thickness.

**Figure 2:**
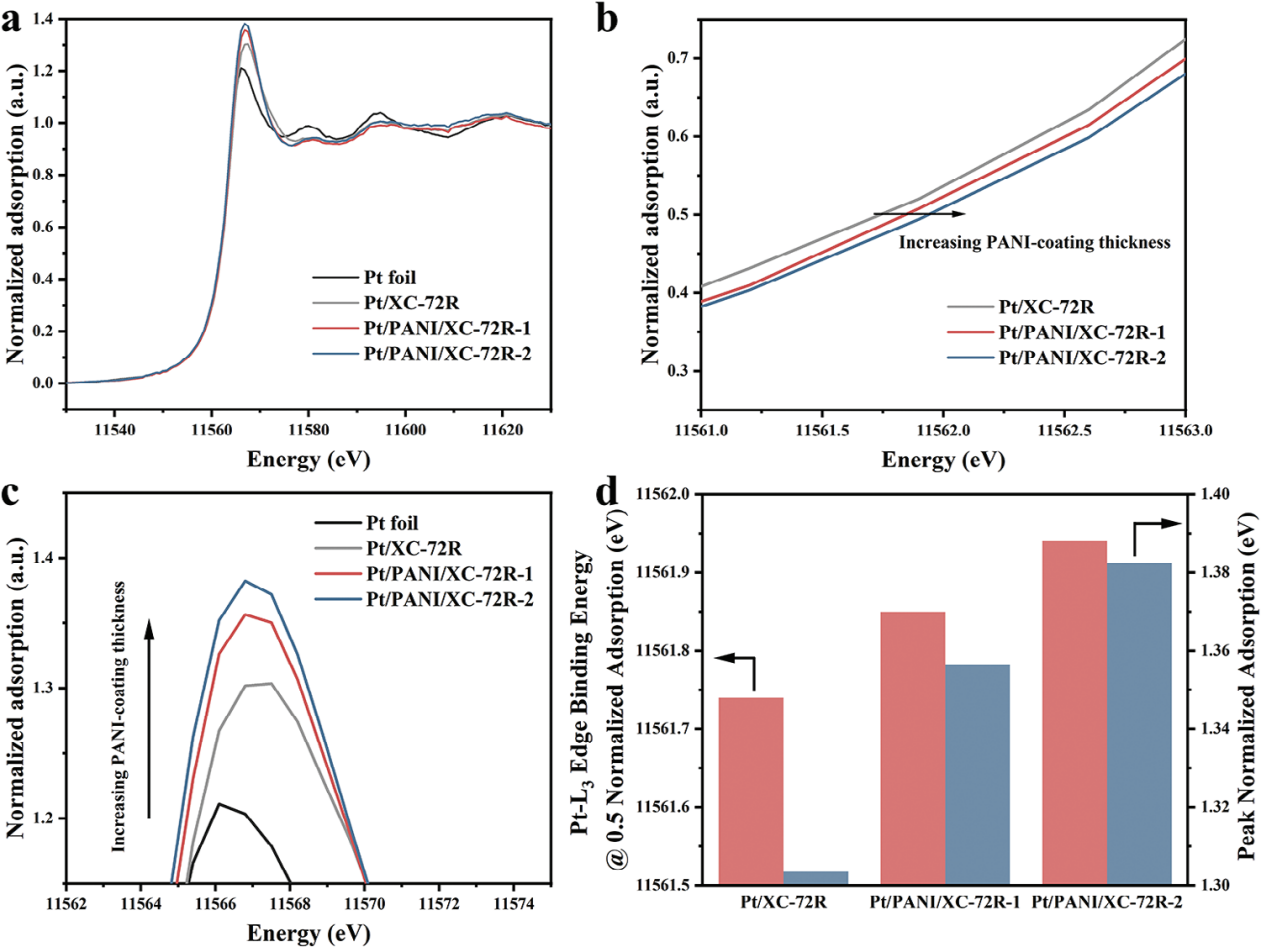
a) XAFS spectrums of the catalysts. b) The XAFS spectrums comparison close to 0.5 normalized adsorption. c) while line (WL) region of XAFS of the catalysts with different PANI‐coating thicknesses and Pt foil. d) Comparison of energy at 0.5 normalized adsorption and peak normalized adsorption for the catalysts with different PANI‐coating thicknesses.

The surface chemical environment and the valence state of Pt catalysts were also investigated by XPS and X‐ray absorption fine structure (XAFS). In terms of Pt 4f signals from XPS results, the binding energy levels of Pt(0) 4f are observed at 71.0 and 74.3 eV, whereas the Pt(II) 4f peaks appear at 72.4 and 75.7 eV. Additionally, the Pt(IV) 4f peaks are discernible at 74.2 and 77.5 eV.^[^ ^27^ ^]^ According to the deconvolution analysis of Pt 4f spectral peaks shown in Figure S8 (Supporting Information), Pt (0) presents a dominant percentage for all uncoated Pt/C and coated Pt/PANI/C samples, indicating a similar Pt reduction during the catalyst synthesis. The Pt 4f_7/2_ and 4f_5/2_ peaks exhibit minimal core‐level shifts for all homemade Pt/C and Pt/PANI/C samples, suggesting the need for higher resolution characterization should be conducted. Hence, the XAFS measurement was conducted, and the results are shown in **Figure** **2**a. Two typical energy absorption positions can be easily compared and highlighted for samples with different PANI‐coating thicknesses. First, the energy corresponding to 0.5 of normalized absorption, also known as L_3_‐edge half‐step energy, indicates a thicker PANI coating results in increased energy at 0.5 of normalized absorption. This observation implies that the PANI‐coating effectively modulates the d‐band electronic state of platinumas shown in Figure 2b. Besides, the peak intensities of while lines (WLs) in the XAFS results of the Pt L_3_ edge, caused by the photoelectron transition from the 2p core to the unoccupied 5d state, reflect the electron density in the Pt 5d orbital.^[^ ^28^ ^]^ The peak intensities follow the same trend as the PANI‐coating coating thicknesses, resulting from the electrons transfer from Pt to PANI groups. As shown in Figure 2c, the thicker PANI‐coating thickness leads to the higher intensity of the WLs peak, implying a more vacant Pt 5d orbital and lower electron density. A direct comparison of energy at 0.5 normalized adsorption and peak normalized adsorption for the catalysts with different PANI‐coating thicknesses is shown in Figure 2d.

**Figure 3:**
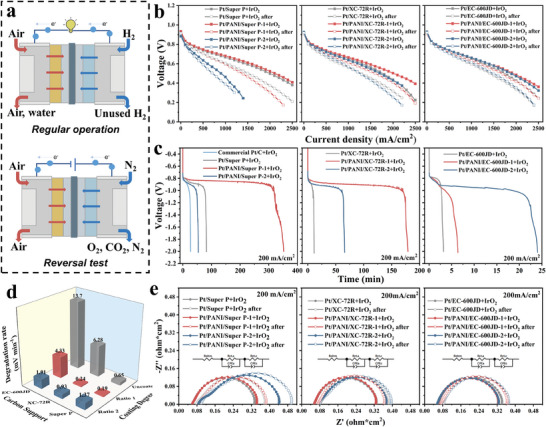
a) Schematic illustration of gases supply during regular operation and reversal test. b) H_2_/air polarization curves, c) reversal tolerance tests, d) voltage degradation rates at 1 A cm^−2,^ and e) EIS test results at 200 mA cm^−2^ of Pt/C+IrO_2_ RTA and Pt/PANI/C+IrO_2_ RTA under fixed flows (0.5 L/1 L).

The electrochemical performance was evaluated using real‐world single‐cell tests. **Figure** **3**a illustrates the gas supply setup during regular operation and reversal tests. The H_2_ was replaced by N_2_ at galvanostatic mode (200 mA cm^−2^) during the reversal test to simulate fuel starvation. To better analyze the effects of different PANI‐coating thicknesses as well as carbon supports, the polarization performance of anodes before and after the reversal test was assessed by conventionally mixing commercial IrO_2_ to fabricate Pt/C+IrO_2_ and Pt/PANI/C+IrO_2_ RTAs (Figure 3b). The results of reversal tests are presented in Figure 3c.

For Super P‐supported catalysts, Pt/PANI/Super P‐1+IrO_2_ RTA displays slightly better initial polarization performance than Pt/Super P+IrO_2_ RTA, with a maximum power density of 1206 and 1180 mW cm^−2^ (0.694 vs 0.684 V at 1 A cm^−2^), respectively. The reversal test of the Pt/PANI/Super P‐1+IrO_2_ RTA demonstrates more than four times longer reversal time than Pt/Super P+IrO_2_ RTA (352 mins vs 82 mins). The OER dominant period was significantly prolonged, indicating that the PANI coating effectively maintains the original catalyst structure, preserving electron and proton transfer pathways by the more uniform and connected Pt nanoparticles network,^[^ ^9^ ^]^ as well as reducing the carbon exposure area, as shown in Figure S4 (Supporting Information). By contrast, the Pt/PANI/Super P‐2+IrO_2_ RTA presents significantly lower polarization performance and greatly decreased reversal time (W_max =_ 619 mW cm^−2^, 0.576 V at 1000 mW cm^−2^, 53 mins) due to the thicker PANI‐coating, even compared to Pt/Super P+IrO_2_ RTA. As observed in Figure S4 (Supporting Information), the thick PANI coating of Pt/PANI/Super P‐2 not only separates the Pt from the carbon support but also disconnects Pt nanoparticles. Moreover, some Pt nanoparticles are inevitably encapsulated by the thick PANI layer, thus deactivating some Pt sites for HOR.^[^ ^29^ ^]^ The Pt nanoparticles wrapped by a thick PANI‐coating layer can also be confirmed by the cyclic voltammetry (CV) tests, as shown in Figure S9 (Supporting Information). The Pt hydrogen under potential deposition (H‐UPD) peaks almost disappear even before the reversal test in terms of the Pt/PANI/Super P‐2+IrO_2_ RTA, suggesting that the Pt nanoparticles cannot be exposed and absorbed. The over‐thick PANI coating also deteriorates the reversal time for Pt/PANI/Super P‐2 since it isolates the contact between the Pt nanoparticles and carbon support or other Pt nanoparticles, cutting off the electron transfer pathway during the OER‐dominating period. As a reference sample, the commercial 47.1% Pt/C with XC‐72R‐based carbon support was also tested, showing the shortest reversal time (27 mins) because of the lower metal coverage and larger carbon exposure area.^[^ ^16^ ^]^

When it comes to the RTAs with XC‐72R‐supported catalysts, the Pt nanoparticles are unable to contact mutually due to the larger surface area of XC‐72R compared with Super P, and the intrinsic property of catalyst support determines the reversal tolerance. Compared with Pt/XC‐72R+IrO_2_ RTA, better polarization performance (1187 vs 1027 mW cm^−2^, 0.689 V versus 0.681 V at 1 A cm^−2^) and more than 16 times prolonged reversal time (179 min vs 11 mins) were obtained for Pt/PANI/XC‐72R‐1+IrO_2_ RTA due to the more uniform Pt distribution and less carbon exposure. In addition, the polarization performance of Pt/PANI/XC‐72R‐2+IrO_2_ RTA is significantly improved compared with Pt/PANI/Super P‐2+IrO_2_ RTA even though it is remains inferior to that of Pt/PANI/XC‐72R‐1+IrO_2_ RTA, as shown in Figure 3b, which is attributed to the larger specific surface area of XC‐72R and thinner PANI‐coating with the same carbon to aniline ratio. The reversal time of Pt/PANI/XC‐72R‐2+IrO_2_ RTA is also prolonged due to the protection of PANI‐coating (65.7 mins), as shown in Figure 3c.

To further validate the broad applicability of the PANI‐coating approach, one typical high‐specific‐surface‐area carbon support, EC‐600JD, was selected to conduct similar tests. Unlike Super P and XC‐72R, the results indicate that Pt/PANI/EC‐600JD‐1+IrO_2_ RTA and Pt/PANI/EC‐600JD‐2+IrO_2_ RTA share comparable and superior initial polarization performance (1120  vs 1097 mW cm^−2^, 0.686 V vs 0.682 V at 1 A cm^−2^) compared to Pt/EC‐600JD+IrO_2_ RTA (1001 mW cm^−2^, 0.679 V at 1 A cm^−2^), as shown in Figure 3b. The improvement is attributed to the significantly higher specific surface area of EC‐600JD (more than 1300 m^2^ g^−1^), hence the PANI‐coating thicknesses of Pt/PANI/EC‐600JD‐1 and Pt/PANI/EC‐600JD‐2 are both thin under different carbon to aniline ratios, leading to similar and enhanced polarization performance. Subsequently, the Pt/PANI/EC‐600JD‐2+IrO_2_ RTA displays enhanced reversal tolerance with a reversal time of 24 mins, significantly longer than Pt/EC‐600JD+IrO_2_ RTA and Pt/PANI/EC‐600JD‐1+IrO_2_ RTA (3 mins and 6 mins).

When the gas flow is adjusted to stoichiometry flow 1.5/2.5, the relationship among three carbon‐supported catalyst RTAs remains consistent, as shown in Figure S10 (Supporting Information). The polarization performance degradation rates are also investigated by measuring the polarization performance at 1 A cm^−2^ after the reversal tests (Figure 3d). For low specific surface area carbon support, such as Super P, 60% metal content is initially high, leading to the formation of a Pt nanoparticle‐connected network on the Super P surface. As reported previously, this network will profoundly enhance reversal tolerance.^[^ ^16^ ^]^ When the PANI‐coating layer is thin, the Pt nanoparticle‐connected network is fully preserved, and the PANI coating can further reduces carbon exposure, resulting in a slower degradation rate. However, the Pt nanoparticle‐connected network is destroyed when the PANI‐coating layer becomes too thick. Even though the carbon exposure is hindered by the PANI‐coating layer, the degradation rate is still increased substantially due to the loss of the Pt nanoparticle‐connected network, which weakens the protective effect of the low specific surface area carbon support by the thick PANI‐coating layer. For medium‐specific surface area carbon XC‐72R, the reversal tolerance enhanced by PANI coating is more significant. Compared with Pt/XC‐72R+IrO_2_ RTA, Pt/PANI/XC‐72R‐1+IrO_2_ RTA, and Pt/PANI/XC‐72R‐2+IrO_2_ RTA display 26 times and 6 times lower degradation rate, suggesting prominent enhancement of reversal tolerance by PANI‐coating. For high specific surface area carbon EC‐600JD, the reversal tolerance enhancement is even more significant since the uncoated sample Pt/EC‐600JD+IrO_2_ RTA displays a fast degradation rate because of the large carbon exposure area as well as low metal coverage, and the PANI‐coating can effectively protect the carbon from external corrosive environment, leading to one order of magnitude lower degradation rate (13.7 vs 1.9 mV min^−1^). A similar conclusion is drawn when calculating the maximum power density degradation rates in mW cm^−2^ min^−1^, as summarized in Figure S11 (Supporting Information).

The electrochemical impedance spectroscopy (EIS) was conducted at galvanostatic mode (200 mA cm^−2^) to further analyze the mass transfer conditions before and after the reversal tests. The impedances were meticulously fitted using the equivalent circuit model shown in Figure 3e and statistically summarized in Table S2 (Supporting Information). The minimal variation in the cathode charge transfer resistance indicates a negligible impact on the polarization performance. Qualitatively, the x intercept in the high‐frequency region corresponds to the ohmic resistance, while the diameter of the semicircle reflects the charge transfer resistance. Typically, the charge transfer resistance of HOR is significantly lower compared to that of ORR due to the faster kinetics. Hence, delineating the HOR charge transfer resistance is challenging unless substantial barrier impedes electron or proton transfer pathways at the anode.^[^ ^30^ ^]^ As depicted in Figure 3e, the initial impedances in terms of Pt/Super P+IrO_2_ and Pt/PANI/Super P‐1+IrO_2_are similar, demonstrating that the thin PANI‐coating layer barely affects the electron and proton transfer. However, the situation changes when the PANI‐coating layer becomes thicker (Pt/PANI/Super P‐2+IrO_2_ RTA). Notably, two considerable semicircles are observed before the reversal test, suggesting significant anode charge transfer resistance and mass transfer obstruction caused by the thick PANI‐coating layer. According to the fitting results of impedances, the anode charge transfer resistance of Pt/PANI/Super P‐2+IrO_2_ RTA is three times higher than that of Pt/PANI/Super P‐1+IrO_2_ RTA (37.44 vs 12.36 mΩ*cm^2^). Besides, the ohmic resistance of Pt/PANI/Super P‐2+IrO_2_ RTA is considerably higher than that of Pt/PANI/Super P‐1+IrO_2_ RTA, implying the over‐thick PANI coating adversely affects electron transfer across the catalyst layer. After the reversal tests, both ohmic and anode charge transfer resistances increase to similar levels for Pt/Super P+IrO_2_ RTA and Pt/PANI/Super P‐1+IrO_2_ RTA, despite the latter experiencing four times the reversal time, confirming the robust protective effect of thin PANI coating layer. A similar trend is observed for XC‐72R‐supported catalyst RTAs, where Pt/XC‐72R+IrO_2_ RTA and Pt/PANI/XC‐72R‐1+IrO_2_ RTA show comparable ohmic and charge transfer resistances before and after the reversal tests, even though Pt/PANI/XC‐72R‐1+IrO_2_ RTA experienced 16 times longer reversal time. The worse polarization performance of Pt/PANI/XC‐72R‐2+IrO_2_ RTA is also reflected in more considerable ohmic and anode charge transfer resistances, compared to Pt/XC‐72R+IrO_2_ RTA and Pt/PANI/XC‐72R‐1+IrO_2_ RTA. For EC‐600JD‐supported catalyst RTAs, the ohmic and anode charge transfer resistances of PANI‐coating samples are slightly lower even for Pt/PANI/EC‐600JD‐2+IrO_2_ RTA, explaining why Pt/PANI/EC‐600JD‐1+IrO_2_ RTA and Pt/PANI/EC‐600JD‐2+IrO_2_ RTA display better polarization performance than Pt/EC‐600JD+IrO_2_ RTA. Besides, the impact of Pt and IrO_2_ loading was also investigated by polarization performance, reversal test, and EIS test, as shown in Figure S12 (Supporting Information). When the Pt and IrO_2_ loadings were doubled, similar trends were observed, with enhanced polarization performance and reversal tolerance for Pt/PANI/Super P‐1+IrO_2_ RTA. The polarization performance difference between Pt/Super P+IrO_2_ RTA and Pt/PANI/Super P‐1+IrO_2_ RTA becomes smaller as 0.2 mg_pt_ cm^−2^ is sufficient for HOR, and more uniform Pt and ionomer distributions can hardly contribute to the polarization performances.^[^ ^31^ ^]^ In addition, as a reference, the Pt/C@PANI is synthesized by coating PANI outside the Pt/C. As shown in Figure S13 (Supporting Information), commercial Pt/C+IrO_2_ RTA displays better polarization performance and reversal tolerance than Pt/C@PANI+IrO_2_ RTA (0.66 V versus 0.63 V at 1 A cm^−2^, 61 mins vs 40 mins), suggesting the external coating of PANI will block the active sites and hinder the mass transfer on the Pt nanoparticle surface.

**Figure 4:**
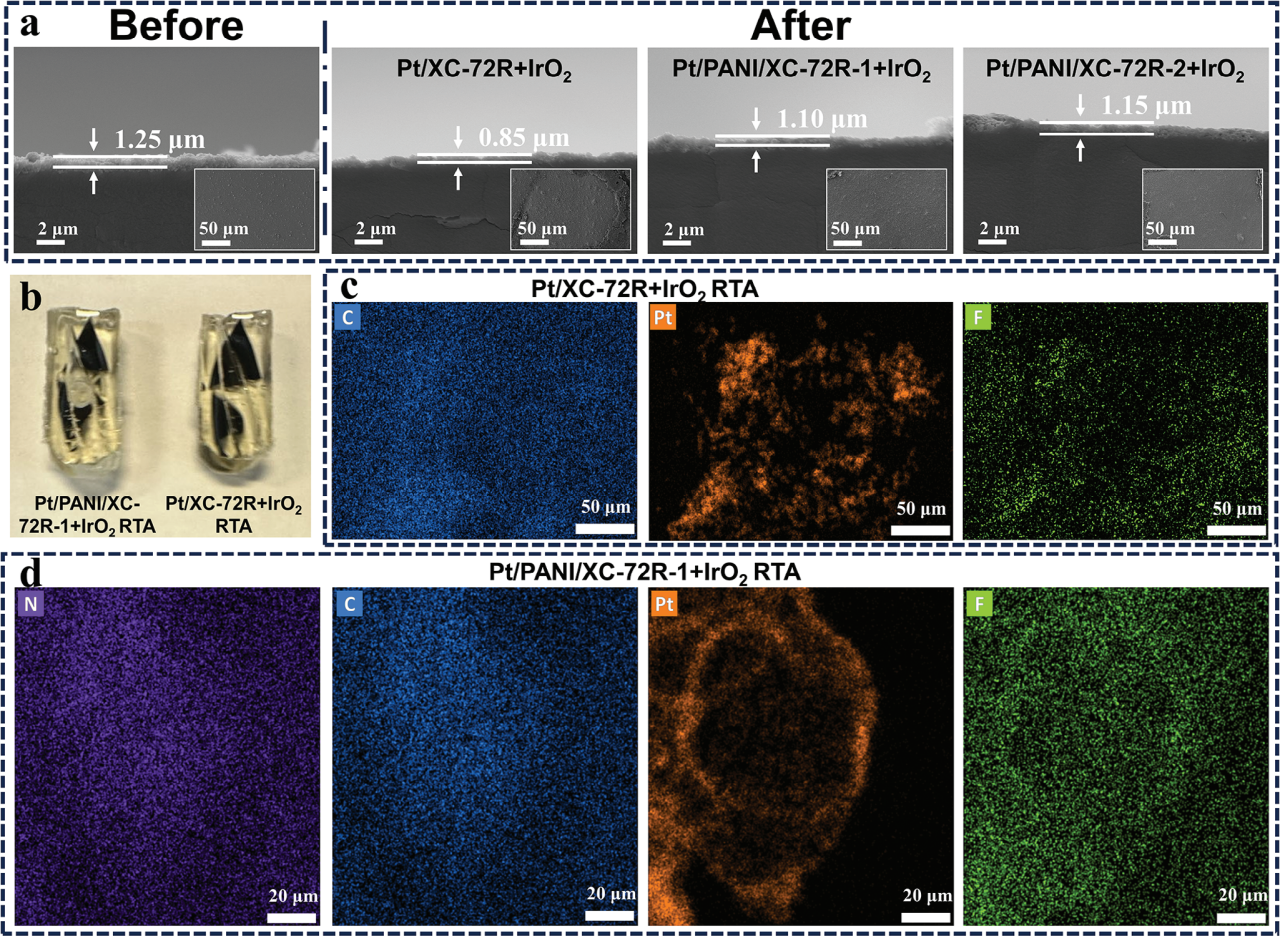
a) SEM images of XC‐72R‐supported catalyst fabricated anodes before and after the reversal tests. b) Resin embedding samples and corresponding EDS mapping of c) Pt/XC‐72R+IrO_2_ RTA and d) Pt/PANI/XC‐72R‐1+IrO_2_ RTA. e) ITC results of three catalyst supports, including association constant Ka, enthalpy of binding, and entropy of binding, respectively.

The morphological characterization of the anode catalyst layer is crucial in understanding the effects of the PANI‐coating layer. To evaluate the anode catalyst layer destruction, the in‐plane and cross‐section scanning electron microscope (SEM) images of the anode catalyst layers were analyzed before and after reversal tests. Specifically, XC‐72R‐supported catalyst‐fabricated RTAs are selected as representative cases to investigate the morphology degradation. As shown in **Figure** **4**a, due to the consistent Pt content (60%) of anode catalysts and the identical loading of Pt and commercial IrO_2_, the initial thickness of anode catalyst layers was approximately 1.25 µm for all XC‐72R‐supported catalyst fabricated RTAs. After the reversal tests, all catalyst layers turn thinner due to carbon corrosion. However, it should be noted that PANI‐coating samples Pt/PANI/XC‐72R‐1+IrO_2_ RTA and Pt/PANI/XC‐72R‐2+IrO_2_ RTA display less catalyst layer thinning and better anode catalyst layer structure retention despite undergoing significantly longer reversal times than Pt/XC‐72R+IrO_2_ RTA, suggesting the enhanced anti‐carbon corrosion propeties and reversal tolerance by PANI‐coating. Furthermore, the catalyst layer surface of Pt/XC‐72R+IrO_2_ RTA becomes rough, and a deep pit is observed due to the serious catalyst support destruction during the reversal test. By contrast, the PANI‐coating anode catalyst layers display a similar smooth surface to the initial reference sample, further proving the ability to boost the anode catalyst layer structure retention of PANI‐coating catalysts.^[^ ^32^, ^33^ ^]^

Meanwhile, the PANI‐coating facilitates a uniform ionomer distribution because of the coulombic interaction between the ionomer and N groups on the carbon support surface.^[^ ^34^, ^35^, ^36^ ^]^ To validate the ionomer distribution in the anode catalyst layer, the Pt/XC‐72R+IrO_2_ RTA and Pt/PANI/XC‐72R‐1+IrO_2_ RTA were embedded by resin (Figure 4b) and subsequently sectioned into ultrathin slices for TEM characterization. The F element distribution obtained by STEM‐EDS mapping without Cs staining can accurately reflect the ionomer distribution.^[^ ^37^ ^]^ As shown in Figure 4c,d, the F element tends to accumulate on the Pt nanoparticles surface in terms of Pt/XC‐72R+IrO_2_ RTA. Differently, the F element in Pt/PANI/XC‐72R‐1+IrO_2_ RTA presents a more homogeneous distribution across the entire catalyst particle surface, instead of aggregating around the Pt nanoparticles surface. This uniform distribution significantly mitigates the uneven spatial distribution of the ionomer, contributing to improved polarization performance. To clarify the interaction between the ionomer and three carbon supports with different PANI‐coating thicknesses, the ITC analysis was conducted to quantify the ionomer‐carbon interaction.^[^ ^38^ ^]^ The adsorption heat was measured by titrating the ionomer into the carbon ink, while the related parameters were calculated by fitting the isotherm to an independent binding model. Meantime, based on the TG results, the average densities were calculated, enabling the extraction of supplementary thermodynamic binding information. This process necessitates certain assumptions regarding ionomer aggregate^[^ ^39^ ^]^ and particle size^[^ ^40^ ^]^ to determine the molar concentration of ionomer and catalyst binding sites for carbon supports (Figures S12 and S13, Supporting Information). As shown in Figure 4e, the association constant Ka is greater for thicker PANI‐coating, suggesting a heightened affinity between the ionomer and PANI layer. Besides, the enthalpic contributions and entropic contributions for binding to PANI‐coating carbon supports follow the same trend as Ka. All results point out that the PANI‐coating carbon supports, which contain the N functional group, strongly attract the negatively charged ‐SO_3_‐ groups of ionomer once they contact, leading to a more uniform ionomer distribution.^[^ ^36^ ^]^

**Figure 5:**
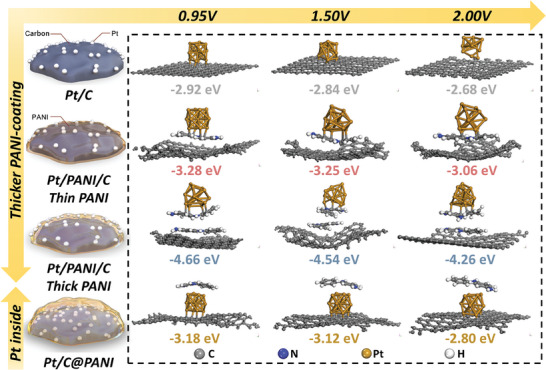
Schematic illustration and atomistic pictures of Pt/C, Pt/PANI/C with a thin PANI layer, Pt/PANI/C with a thick PANI layer, and Pt inside Pt/C@PANI catalysts with the binding energy of the calculated structures as a function of three applied potentials (0.95, 1.50, and 2.00 V).

To better understand the origins of structure stability of PANI‐coating carbon‐supported catalysts, a series of first‐principles calculations were conducted based on the DFT^[^ ^41^, ^42^ ^]^ with generalized gradient approximation (GGA) in the form of Perdew‐Burke‐Ernzerhof (PBE) function for exchange‐correlation potential.^[^ ^43^, ^44^ ^]^ Three typical potentials were selected to represent the thermodynamic stability: regular operation potential (0.95 V) and high potential during the cell reversal (1.50 and 2.00 V). The study considered four cases with different PANI‐coating scenarios:(**Figure** **5**), a) Pt/C: This case started with a periodic 8 × 8 graphene surface model with 64C atoms, onto which a Pt_13_ nanoparticle was attached to predict the interaction energetics between Pt nanoparticles and the graphene surface; b) Pt/PANI/C thin PANI: One‐layer PANI was placed between Pt and graphene to simulate the situation when a thin PANI layer is coated on the graphene surface; c) Pt/PANI/C thick PANI: Two‐layer PANI was placed between Pt and graphene to simulate a thick PANI layer coated on the graphene surface and (d) Pt/C@PANI: Pt nanoparticles were placed between PANI and graphene to simulate the situation when PANI was deposited after Pt nanoparticles.

The binding energy of each case was calculated using the formula:

(4) $$\begin{array}{ccc} \mathrm{Binding}\;\mathrm{energy} & = & E\left(\mathrm{Pt}+\mathrm{PANI}+\mathrm{graphene}\right)-E\left(\mathrm{Pt}\right)\\ & & -E\left(\mathrm{PANI}+\mathrm{graphene}\right) \end{array}$$

The results are shown in Figure 5 and Figure S16 (Supporting Information). The binding energy is used to predict structure stability under high potential from a thermodynamic perspective, where a stronger binding energy increases the barrier again structure collapse, carbon corrosion, and Pt detachment.^[^ ^25^ ^]^ As expected, the binding energy decreases when the potential increases from 0.95 to 2.00 V, indicating that higher potentials will deteriorate the structural stability. In addition, the PANI‐coating cases show stronger binder energies than uncoated Pt/C cases, and the thicker PANI layer can further increase the binder energy, explaining the structure protection and better durability of PANI‐coating catalysts under high potential. It should be noted that thees calculations only reflect the thermodynamic properties instead of the kinetics properties, which cannot represent the adsorption and desorption of reactants. Interestingly, the binder energies become significantly smaller when the PANI is coated outside the Pt/C (Pt/C@PANI), which is in excellent agreement with the experimental observations.

**Figure 6:**
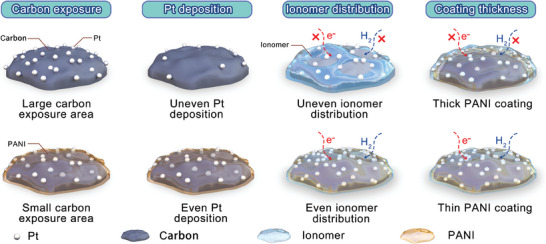
Schematic illustration of optimization mechanisms of Pt/PANI/C.

**Figure** **6** illustrates the optimization mechanisms of PANI‐coating anode catalysts. The advantages of PANI‐coating can be divided into three parts. First, the PANI‐coating can decrease the direct carbon exposure to the outer high potential and corrosion environment during the cell reversal, which is strongly related to the reversal tolerance. The corrosion resistance of PANI is significantly stronger than that of carbon, and the PANI acts as a sacrificial and protection layer, maintaining the original catalyst layer structure during reversal destruction. Besides, it has been previously proven that the electrons transfer from Pt toward π‐conjugated systems of the aromatic ring facilitates a strong interaction between Pt atoms and PANI, leading to the N‐groups acting as nucleation centers for Pt and high dispersion of Pt nanoparticles on the PANI surface,^[^ ^45^ ^]^ leading to a higher Pt utilization.^[^ ^46^ ^]^ This phenomenon is confirmed experimentally and universally by the TEM characterization of different carbon supports with the same synthesis processes and conditions. In addition, the introduction of a PANI‐coating layer also affects the spatial distribution of ionomer when fabricating electrodes. The uneven ionomer distribution results in the insufficient proton transfer pathway in the ionomer‐deficient area and increased local gas transport resistance in the ionomer‐sufficient area.^[^ ^47^, ^48^ ^]^ Experimentally, the F element distribution of the PANI‐coating catalyst layer acquired by EDS mapping and ITC analysis validate the optimized ionomer distribution. The homogeneous ionomer distribution is attributed to the coulombic interaction with negatively charged ionomer side chains, such as ─SO_3_^−^. Furthermore, the adjustment of PANI‐coating layer thickness also plays a vital role in optimizing polarization performance and reversal tolerance. When the PANI‐coating layer is too thick, the Pt nanoparticles are become encapsulated by the PANI layer. Under this circumstance, the hydrogen cannot contact Pt nanoparticles, while the loss of electron and proton conductivity caused by the PANI layer is also crucial, leading to the disappearance of the triple‐phase boundary as well as the active sites of HOR. It can be reasonably inferred that there is an optimized PANI‐coating thickness threshold for carbon supports, which should be close to the diameter of the Pt nanoparticles. This optimal thickness achieves the dual goals of protecting the PANI‐coating layer while maintaining contact between Pt nanoparticles and the carbon support, as well as exposure to hydrogen, ensuring smooth electron and gas transport. This phenomenon can be experimentally validated, as demonstrated by the XC‐72R‐related catalyst results. The polarization performance increases when the PANI‐coating is thinner (Pt/PANI/XC‐72R‐1) but decreases when the PANI‐coating thickness continues to increase (Pt/PANI/XC‐72R‐2). For Pt/PANI/XC‐72R‐1, the thickness of the PANI layer is approximately 3 nm, based on TEM observations, which is close to the diameter of Pt nanoparticles (3–4 nm). In this case, the PANI layer enhances both polarization performance and reversal tolerance. However, when the coating thickness increases to 11.5 nm (Pt/PANI/XC‐72R‐2), polarization performance declines, and the first reversal time is shortened, indicating increased resistance to gas and electron transport.

## 3 Conclusion

In this contribution, we synthesized PANI‐coating catalysts (Pt/PANI/C) by a facile in situ polymerization of aniline on commercial carbon support surfaces. Compared to their uncoated counterparts, the PANI‐coating catalyst fabricated RTAs demonstrated enhanced polarization performance and improved reversal tolerance. The improved polarization performance is attributed to the more uniform Pt deposition and ionomer distribution, facilitated by N‐groups acting as nucleation centers and the coulombic interactions. Regarding better reversal tolerance, the PANI‐coating results in a reduced carbon exposure area, decelerating the carbon corrosion rate. Specifically, the MEA with Pt/PANI/XC‐72R‐1+IrO_2_ RTA presents 179 mins reversal time with a degradation rate of 0.24 mV min^−1^ at 1 A cm^−2^, 16 times longer and 26 times lower than the MEA with uncoated Pt/XC‐72R+IrO_2_ RTA. However, the enhancements disappear when the PANI‐coating became excessively thick as the Pt nanoparticles were encapsulated. DFT calculations confirmed that the PANI‐coating layer thermodynamically enhance the structure stability under high potential, which firmly explains the benefits of PANI‐coating. This work provides a facile, practical, and scalable approach to developing high‐performance and durable anodes, emphasizing the importance of catalyst support in the fuel cell community and contributing to the broader commercialization of PEMFCs.

## 4 Experimental Section

### Synthesis of PANI/C Support and Pt/PANI/C

The PANI/C was prepared by in situ polymerization of aniline on commercial carbon supports, including Super P (TIMICAL), XC‐72R (Cabot), and EC‐600JD (Ketjenblack) with two different aniline‐to‐carbon mass ratios. Typically, for recipe 1 (carbon:aniline = 1.4:1), 21 mg carbon support was dispersed into 100 mL ethanol instead of water since pure carbon supports are highly hydrophobic. Then, 2.72 mL of concentrated sulfuric acid and 14.6 µL of aniline monomer (Aladdin, 99.5%) were added, followed by ultrasonic blending for 30 min and stirring overnight to obtain a homogeneous dispersion. After that, 100 mL of 0.5 m H_2_SO_4_ aqueous solution containing 36.75 mg of (NH_4_)_2_S_2_O_8_ (ammonium peroxydisulfate, APS, Aladdin, 99.99%) was added dropwise with vigorous stirring (the molar ratio of APS to aniline was controlled at 1:1) under an ice bath for 10 h. The mixture was filtered, rinsed with distilled water, and dried under vacuum at 80 °C overnight. Following this procedure, catalysts with recipe 2 (carbon:aniline = 1.4:2) were also prepared.

To synthesize Pt/PANI/C, a straightforward ethylene glycol reduction method was utilized. Initially, 12 mg of Pt precursor solution (H_2_PtCl_6_·xH_2_O, Sigma Aldrich) and 8 mg of PANI/C support were introduced into 250 mL of ethylene glycol (60% Pt content). The size of Pt nanoparticles was regulated by the concentration of Pt precursor. Following stirring for 8 h, the solution was heated to 160 °C for 8 h to facilitate the reduction of platinum. Once cooling to ambient temperature, 0.5 m H_2_SO_4_ was incorporated, and pH was adjusted to 1 to facilitate the adsorption of platinum nanoparticles onto the PANI/C surface. The catalysts were then vacuum‐filtered and washed with sufficient hot deionized water. Finally, Pt/PANI/C was obtained by drying at 60 °C overnight. The Pt/C was prepared for comparison by depositing Pt on commercial carbon supports following the same processes. Based on different aniline‐to‐carbon ratios, the as‐prepared catalysts were donated as uncoated samples (Pt/Super P, Pt/XC‐72R, and Pt/EC‐600JD), recipe 1 samples (Pt/PANI/Super P‐1, Pt/PANI/XC‐72R‐1, and Pt/PANI/EC‐600JD‐1), and recipe 2 samples (Pt/PANI‐Super P‐2, Pt/PANI/XC‐72R‐2, and Pt/PANI/EC‐600JD‐2). The Pt/C@PANI synthesis followed the same processes, except the PANI polymerization is conducted directly on the Pt/C surface.

### Characterizations

The surface area of the catalyst was evaluated by BET analysis utilizing nitrogen isotherms (Micromeritics ASAP 2460). XRD was conducted using a Rigaku Smartlab 3 kW diffractometer employing a Cu Kα radiation source at 40 kV and 30 mA, with a scan rate of 8° min^−1^. XPS was conducted by Thermo Fisher ESCALAB Xi+ using monochromated Al Kα radiation. The ultrathin sectioning was conducted by Leica EM UC7. TEM images were captured using Talos F200X G2. TG analysis was carried out using a NETZSCH STA 449 F3 from 30 to 1000 °C under N_2_ at a heating rate of 10 °C min^−1^. TG‐IR analysis was performed using an Invenior‐STA449F5 under N_2_, with a wavenumber range from 500 to 4000 cm^−1^ and a temperature range from 30 to 800 °C. XAFS spectroscopy was conducted via the RapidXAFS HE Ultra (Anhui Absorption Spectroscopy Analysis Instrument Co., Ltd.) in transmission mode, operating at 20 kV and 20 mA. The Pt analysis utilized a Si (771) spherically bent crystal analyzer with a radius of curvature of 500 mm.

### Electrode Preparation and Performance Testing

Membrane electrode assembly (MEA) was prepared using a catalyst‐coated membrane (CCM) with an active area of 4 cm^2^ and gas diffusion layers (MB30, Avcarb). The anode catalyst ink was made by the ultrasonication of as‐prepared Pt/C or Pt/PANI/C, D520 Nafion ionomer, and 1:1 water/isopropanol solvent for 90 mins. The cathode catalyst ink was treated similarly using a commercial Pt/C (47.1%, TEC10EA50E, Tanaka) catalyst. The mass content of ionomers in the anode and cathode catalyst layers was controlled at 25 and 30 wt.%, respectively. Subsequently, the dispersed ink was sprayed onto both surfaces of the membrane (Nafion NR211, Chemours), while the Pt loading was controlled at 0.1 and 0.4 mg_Pt_ cm^−2^ for the anode and cathode, respectively. The loading of IrO_2_ was kept at 0.05 mg_Ir_ cm^−2^ for all the MEA samples. Two Teflon sheets with a thickness of 0.15 mm were utilized as sealing gaskets to ensure gas‐tight conditions during fuel cell tests operated on a Hephas HTS‐125. During the cell reversal test, nitrogen and air were supplied to the anode and cathode at flow rates of 0.4^1^ and 0.2 L min^−1^, without back pressure. A galvanostatic control at 0.2 A cm^−2^ was administered, and the cell voltage was monitored by the Solartron Energylab XM until it reached −2.0 V, at which point the cell would shut down. The polarization performance, cyclic voltammetry (CV), and electrochemical impedance spectroscopy (EIS) tests were carried out before and after the cell reversal experiments. The EIS was tested at 200 mA cm^−2^ with 100% relative humidity H_2_/air, scanning the frequency from 100 kHz to 0.1 Hz. The CV was carried out under 100% relative humidity N_2_/H_2_ with 0.1 L min^−1^ and on back pressure, scanning the voltage range from 100 to 1000 mV (50 mV s^−1^). To assess the polarization performance, two different flow rate conditions were examined (fixed flows 0.5/1 L min^−1^ and stoichiometry 1.5/2.5) for comparative purposes. The fixed flows test ensured sufficient gas supply to address mass transfer issues, while the stoichiometry flow test provided a realistic condition to evaluate actual performance.

### ITC Measurement

Binding experiments were performed by Microal PEAQ‐ITC. Briefly, the ionomer samples were diluted to 2.75 mg mL^−1^ by deionized water. The ionomer was titrated into a cell with 0.5 mg mL^−1^ carbon supports in deionized water. First, a 0.4 µL ionomer aliquot was injected. Then twelve 3 µL of ionomer aliquots were injected. Baseline measurements were taken (ionomer in H_2_O, H_2_O in carbon support, and H_2_O to H_2_O) to compensate for the heat of dilution/mixing in the outcomes. Subsequently, the area under each injection peak was calculated, and the corresponding heats were fitted to a distinct binding isotherm. The association constant was derived from the model fitting, and error margins were determined from the confidence interval of the fit.

### Computational Methods

The first‐principles calculations were conducted by the density functional theory (DFT) with generalized gradient approximation (GGA) in the form of the Perdew–Burke–Ernzerhof (PBE) function for exchange‐correlation potential. The algorithm was used with Damped MD. The Tkatchenko Scheffler (TS) method was used for DFT‐D dispersion correction. The convergence tolerances for geometric optimization were set to 1.0 × 10^−5^ eV atom^−1^ on the energy, 0.03 eV A^−1^ on the force, 0.0001 nm on the displacement, and 1.0 × 10^−6^ eV atom^−1^ on the SCF, respectively. The zero‐point energy correction was accounted for all of the calculations.

## Conflict of Interest

The authors declare no conflict of interest.

## Supporting information



Supporting Information

## Data Availability

The data that support the findings of this study are available from the corresponding author upon reasonable request.
