# Supplementary material for: A Universal Strategy to Enhance Polarization Performance and Anode Reversal Tolerance by Polyaniline‐Coated Carbon Support for Proton Exchange Membrane Fuel Cells
Source: Adv Sci (Weinh). 2024 Oct 1;11(44):2407570. doi: 10.1002/advs.202407570 (PMC11600206; doi:10.1002/advs.202407570)
Supplement: Supplementary file 1 — Supporting Information [file ADVS-11-2407570-s001.docx]

**Supporting Information**

**A Universal Strategy to Enhance Polarization Performance and Anode Reversal Tolerance by Polyaniline-Coated Carbon Support for Proton Exchange Membrane Fuel Cells**

Zheng Li^a, b, c^, Yongbiao Mu ^b,c^, Qing Zhang^b,c^, Cailin Xiao^b,c^, Yuting Jiang^a, b, c^, Lei Du^d, *^, Siyu Ye^d,e*^, Tianshou Zhao^a, b, c*^, Lin Zeng^b, c*^

a Department of Mechanical and Aerospace Engineering, The Hong Kong University of Science and Technology, Clear Water Bay, Kowloon, Hong Kong, China

b Shenzhen Key Laboratory of Advanced Energy Storage, Department of Mechanical and Energy Engineering, Southern University of Science and Technology, Shenzhen 518055, China

c SUSTech Energy Institute for Carbon Neutrality, Southern University of Science and Technology, Shenzhen 518055, China

d Huangpu Hydrogen Energy Innovation Centre, School of Chemistry and Chemical Engineering, Guangzhou University, Guangzhou, 510006, Guangdong, China

e SinoHykey Technology Company Ltd., Guangzhou, 510760, Guangdong, China


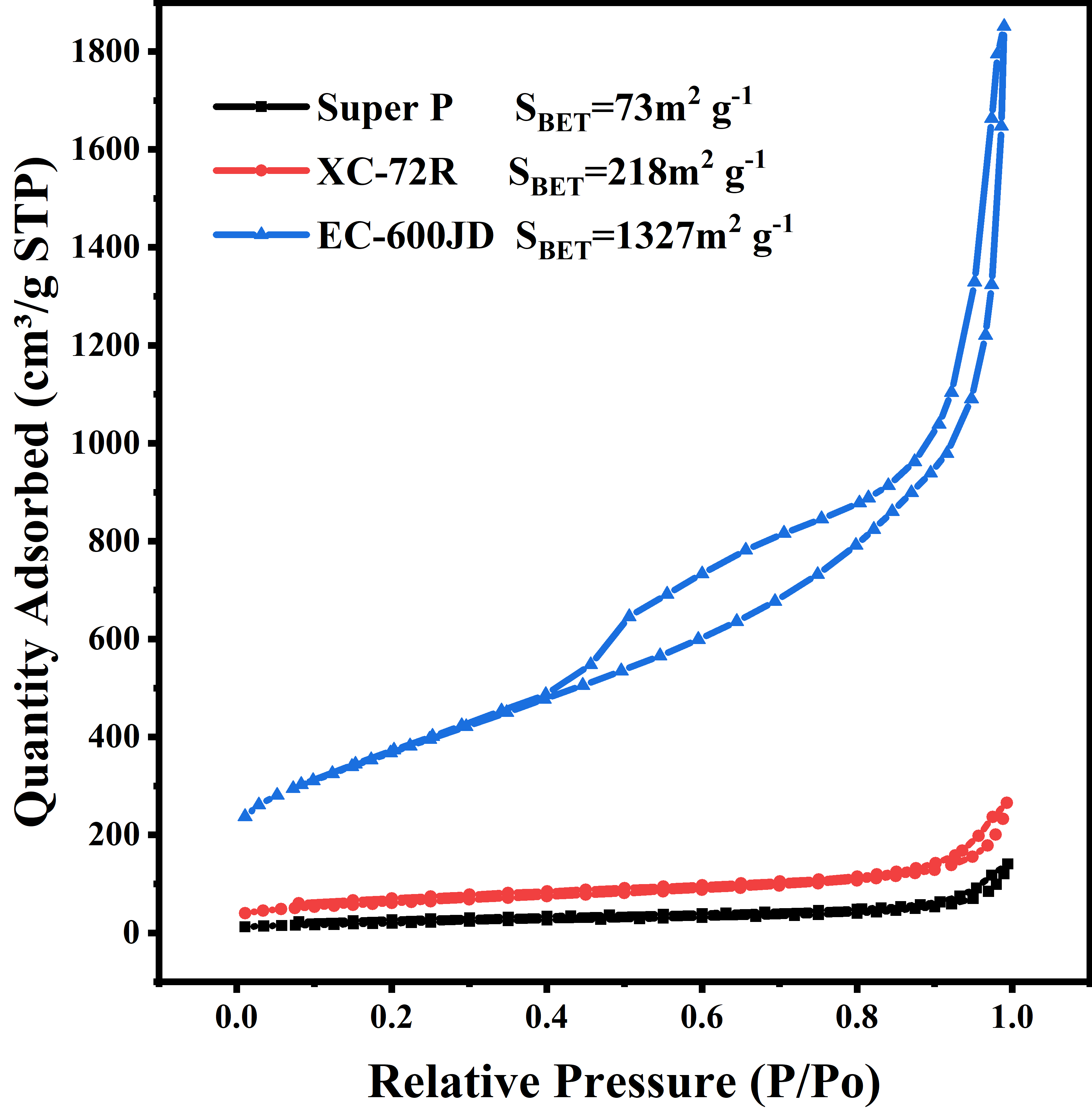


Figure S1. N_2_ adsorption and desorption isotherms of Super P, XC-72R and EC-600JD carbon supports.


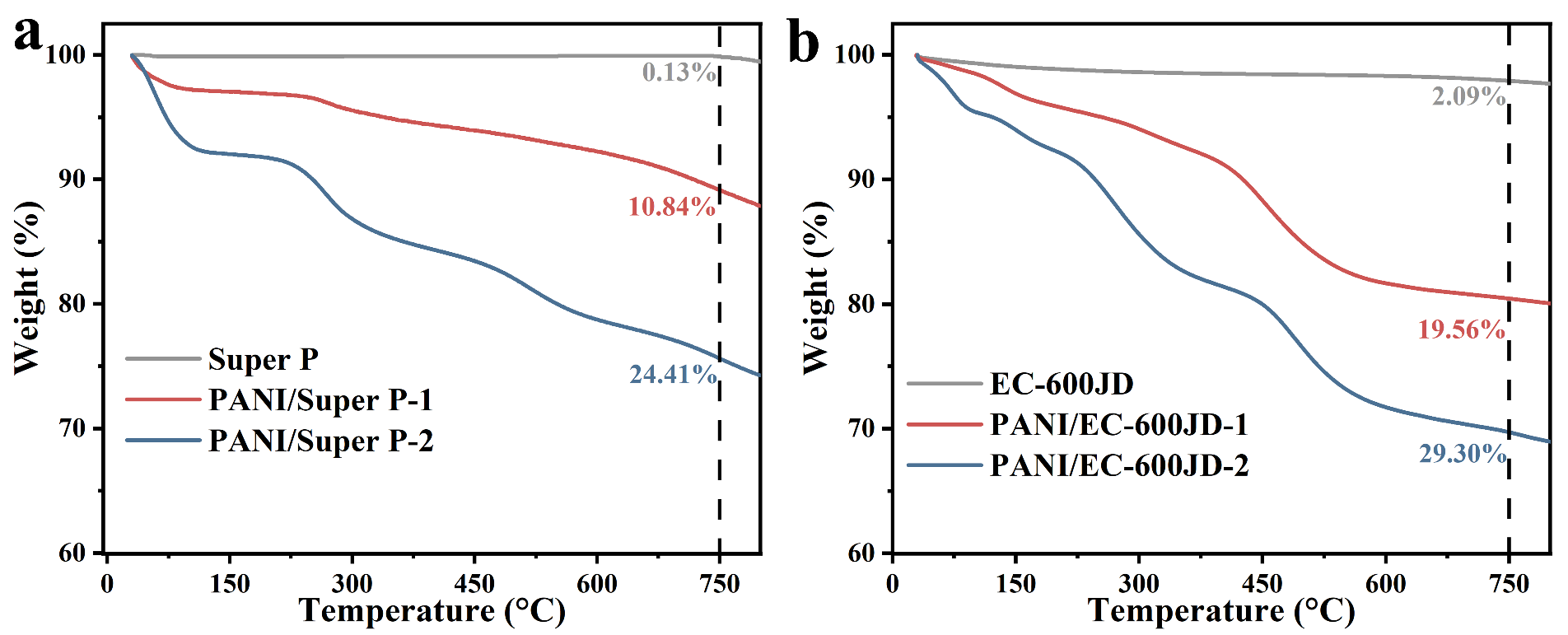


Figure S2. TGA analysis of (a)Super P, PANI/Super P-1 and PANI/Super P-2; (b) EC-600JD,PANI/EC-600JD-1 and PANI/EC-600JD-2.


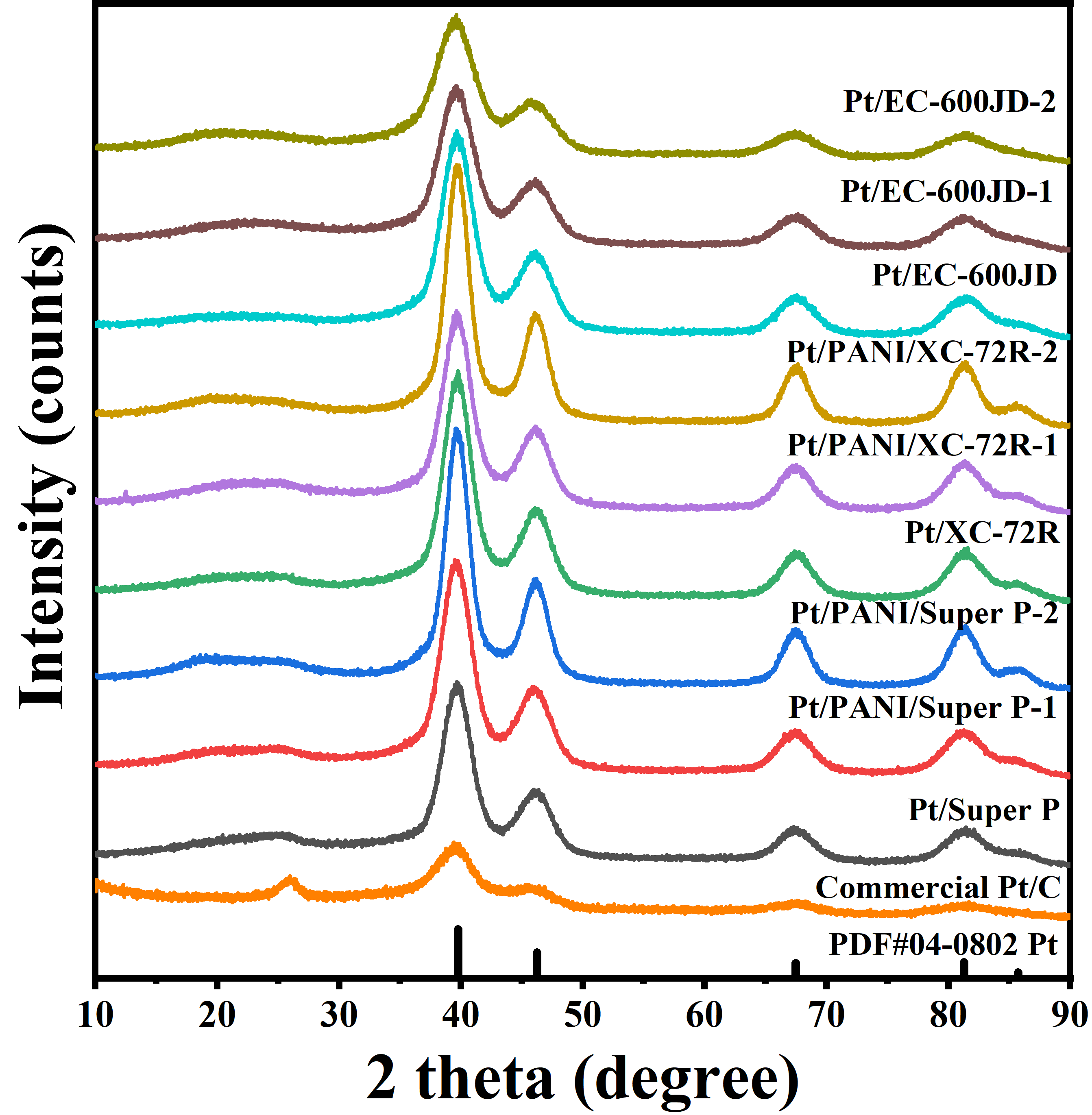


Figure S3. XRD patterns of all Pt/C and Pt/PANI/C samples.


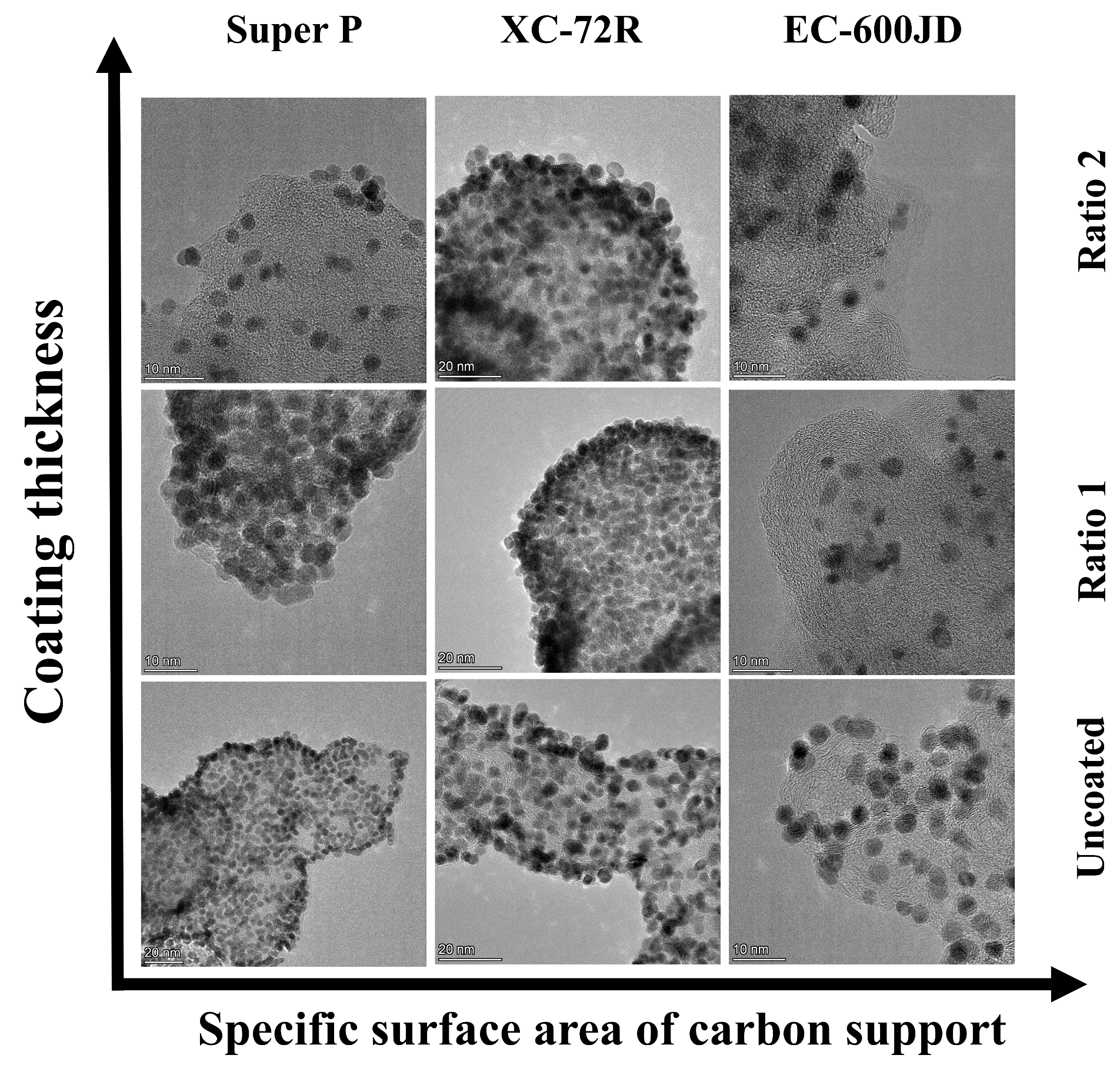


Figure S4. TEM images of Pt/C and Pt/PANI/C with different carbon supports and carbon to aniline ratios.


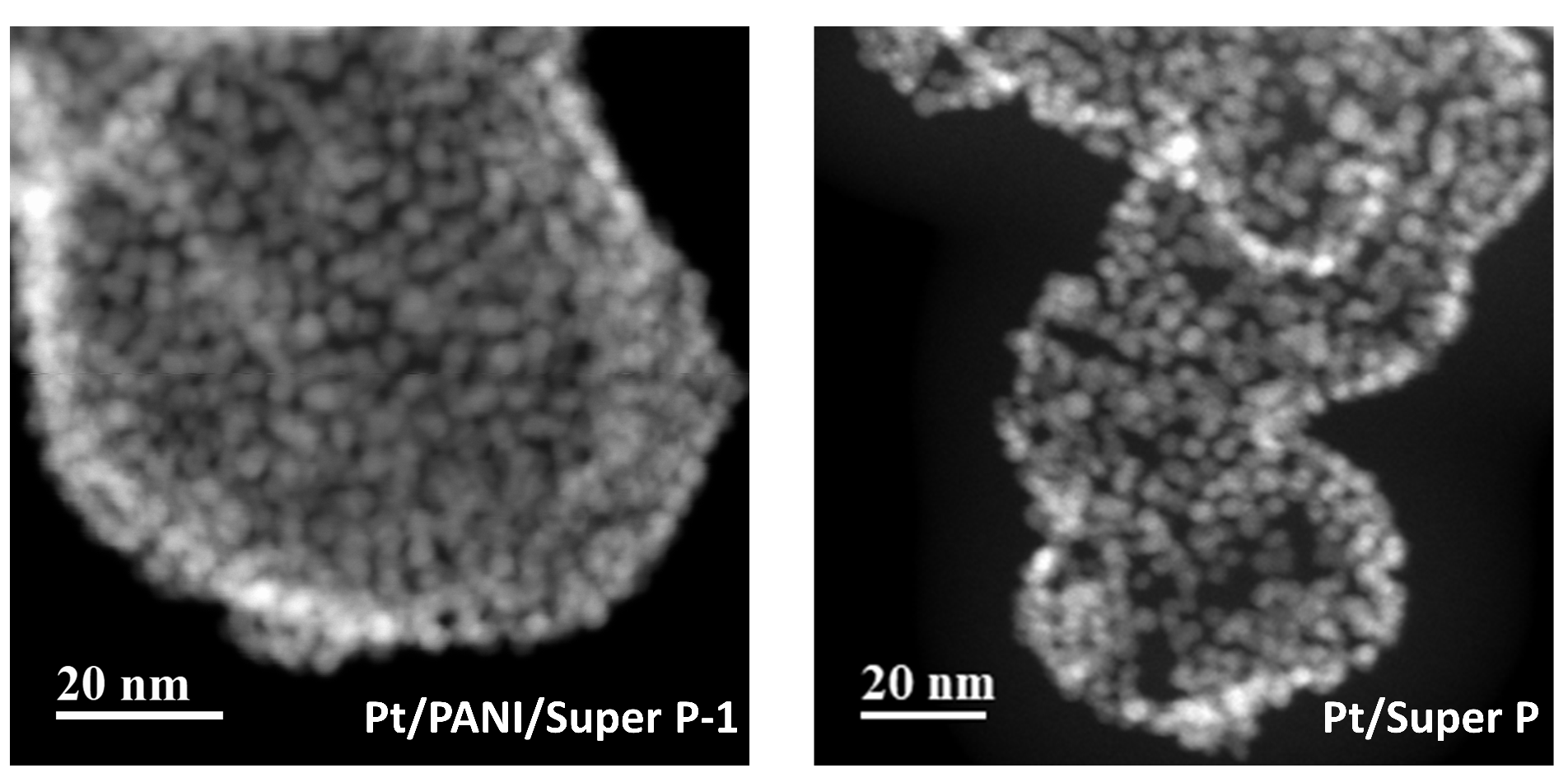


Figure S5. TEM images of Pt/PANI/Super P-1and Pt/Super P.


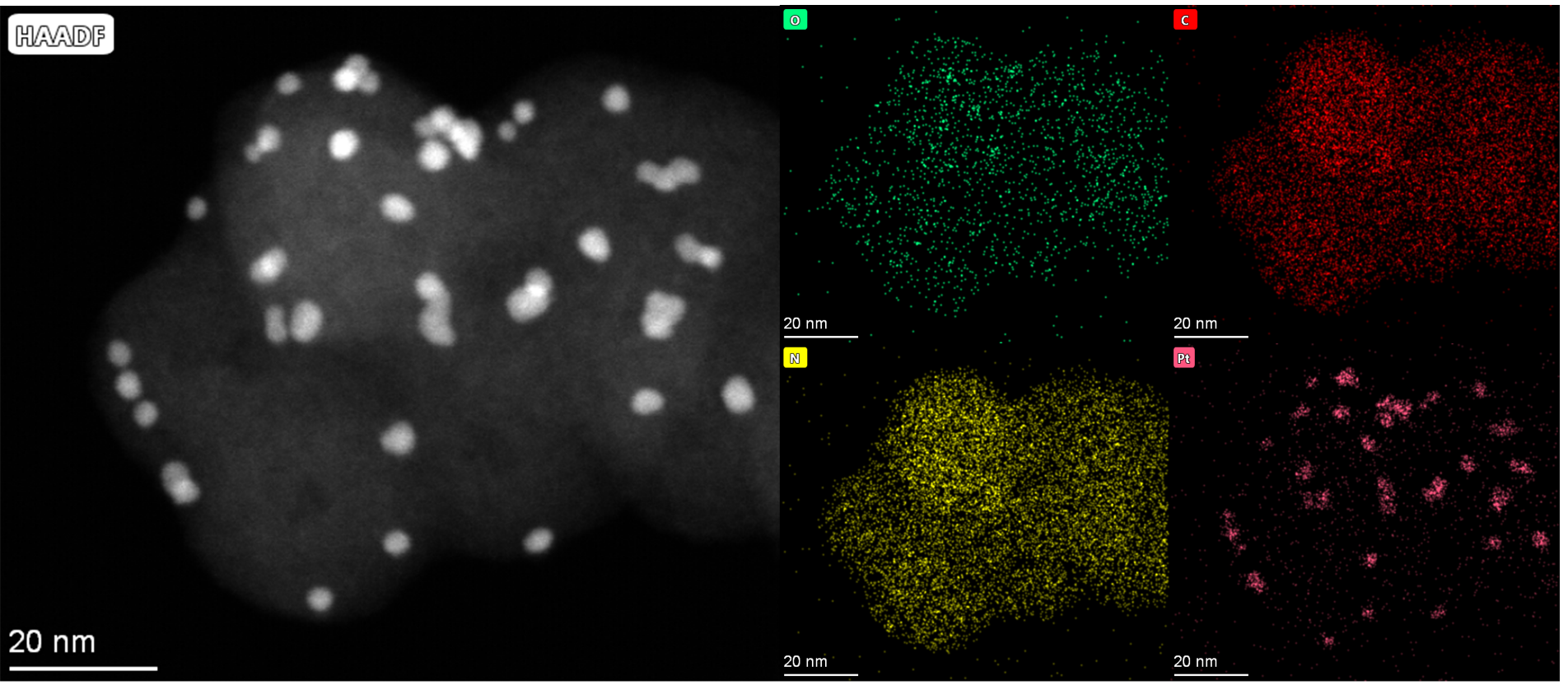


Figure S6. HAADF image and EDS mapping of Pt/PANI/EC-600JD-1.


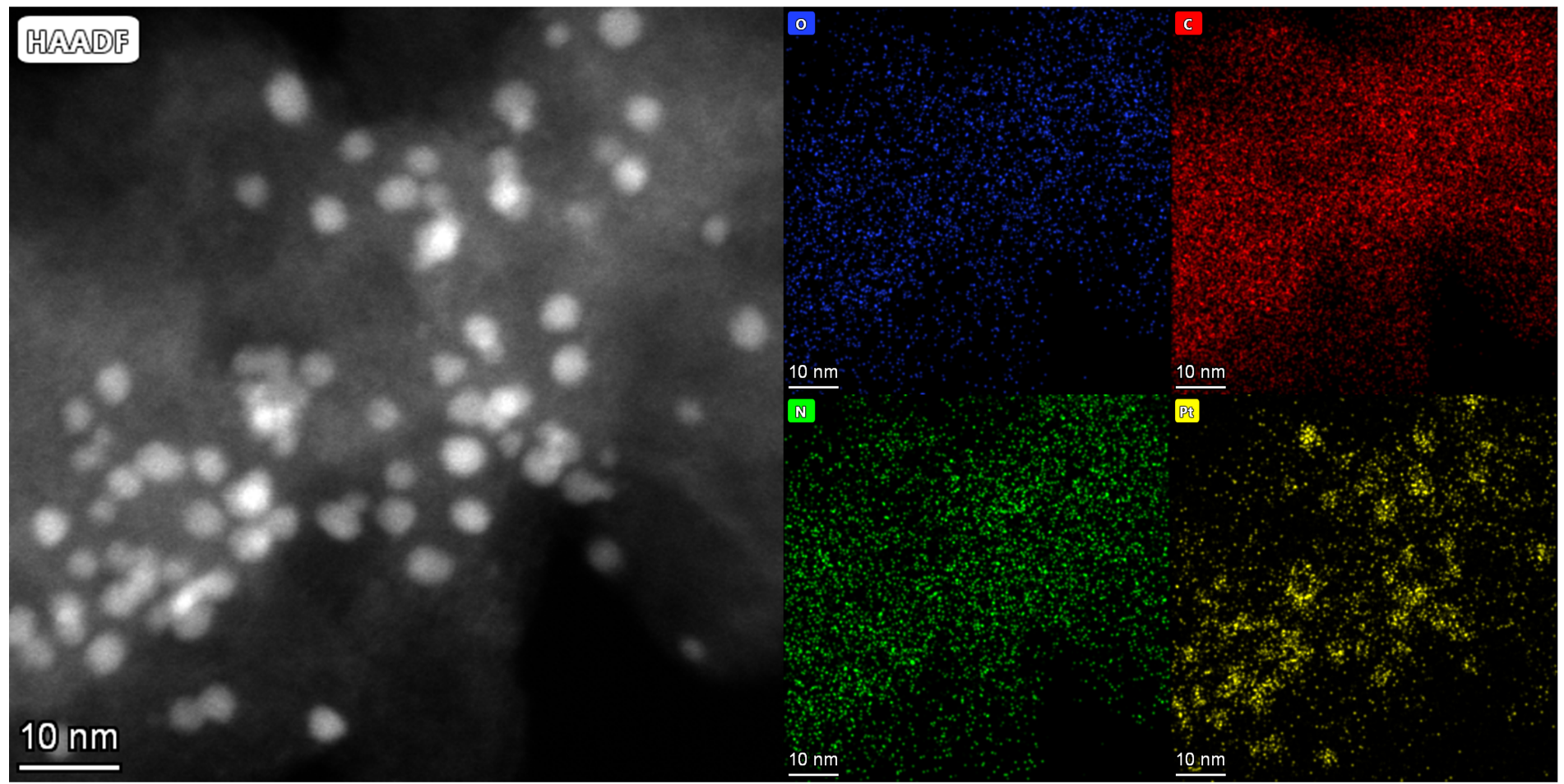


Figure S7. HAADF image and EDS mapping of Pt/PANI/EC-600JD-2.


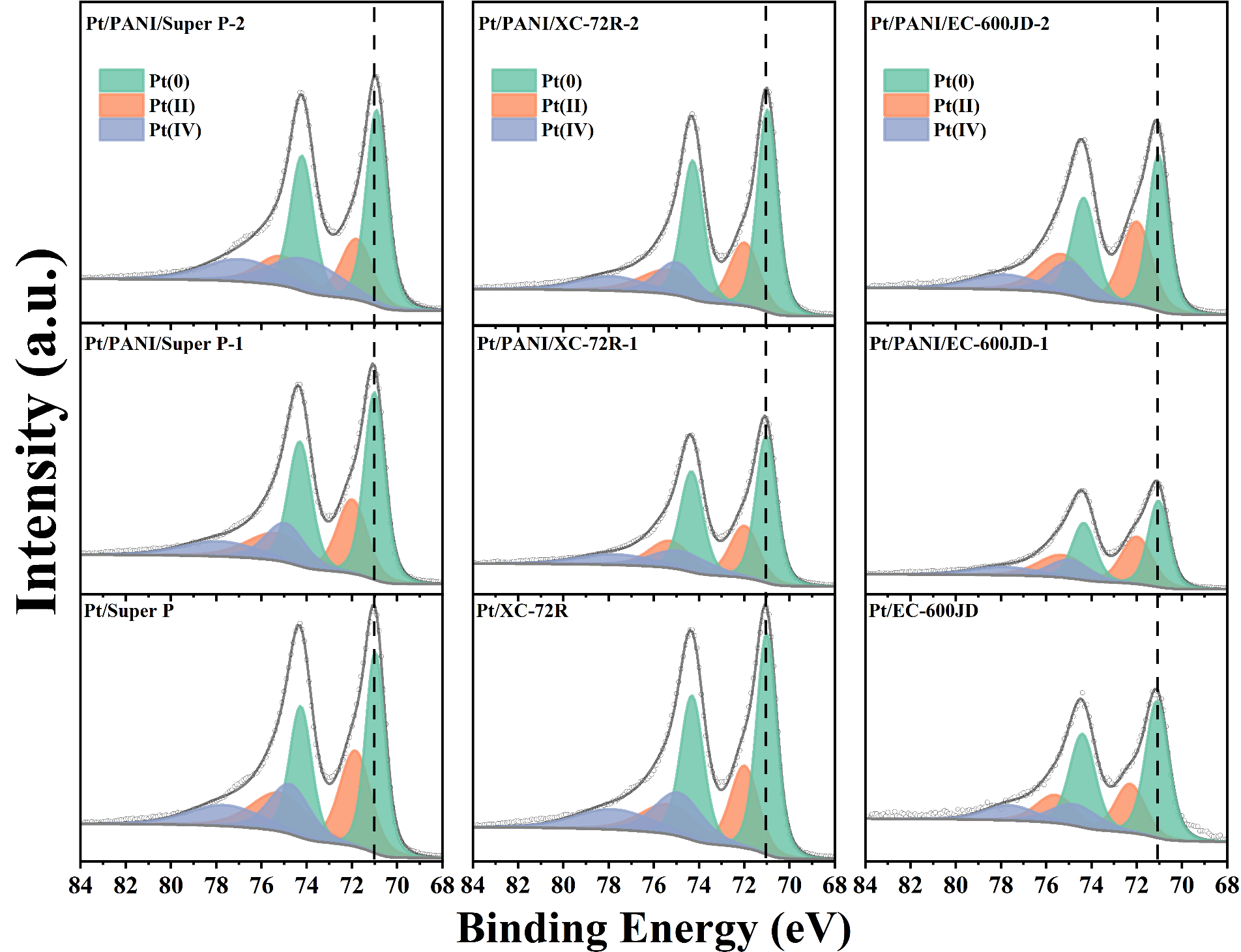


Figure S8. XPS Pt 4f spectra of all Pt/C and Pt/PANI/C samples.


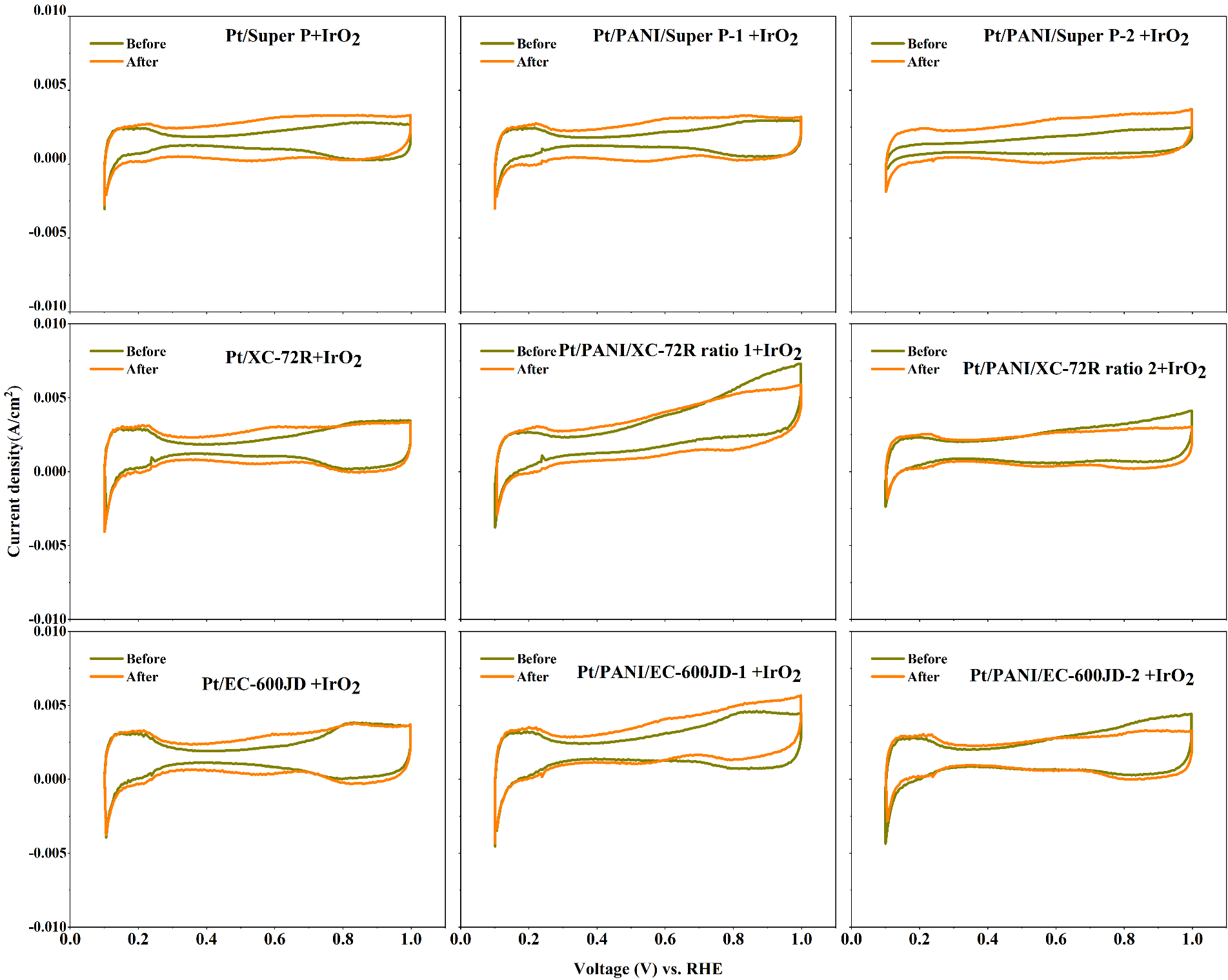


Figure S9. Cyclic voltammetry (CV) curves of Pt/C +IrO_2_ RTAs and Pt/PANI/C +IrO_2_ RTAs before and after reversal tests.


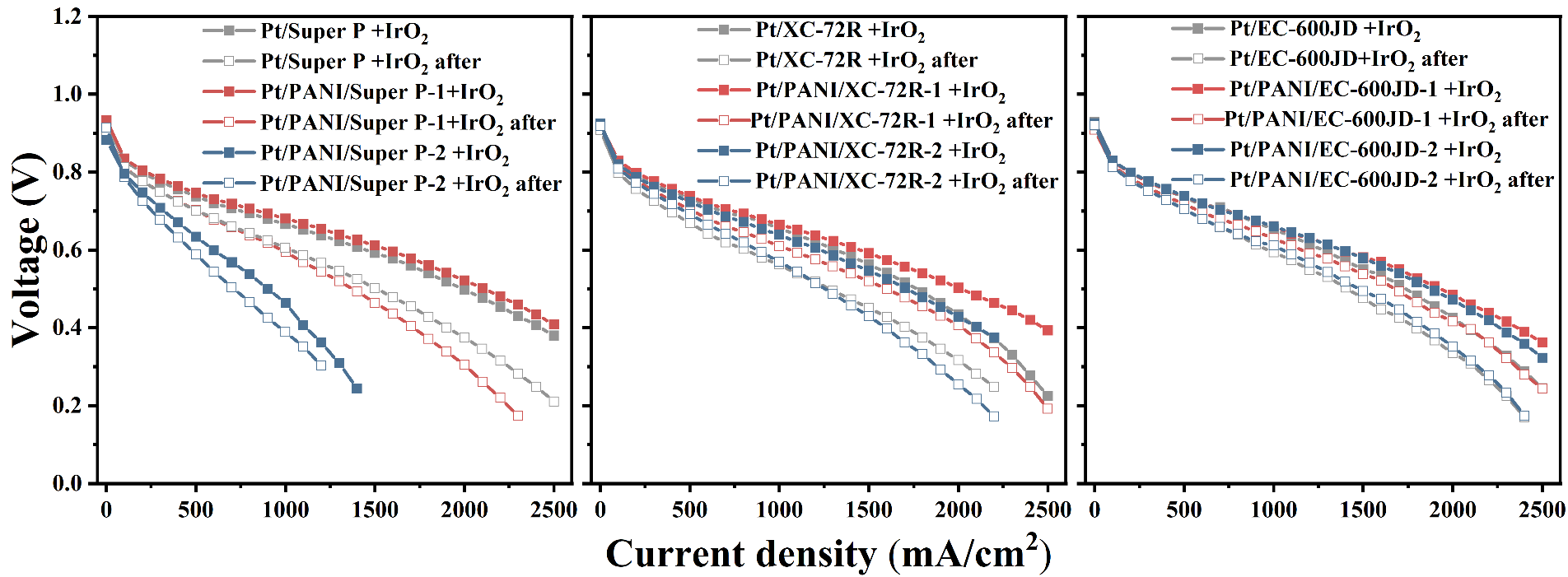


Figure S10. Polarization curves of Pt/C +IrO_2_ RTA and Pt/PANI/C +IrO_2_ RTA under stoichiometry flows (1.5/2.5).


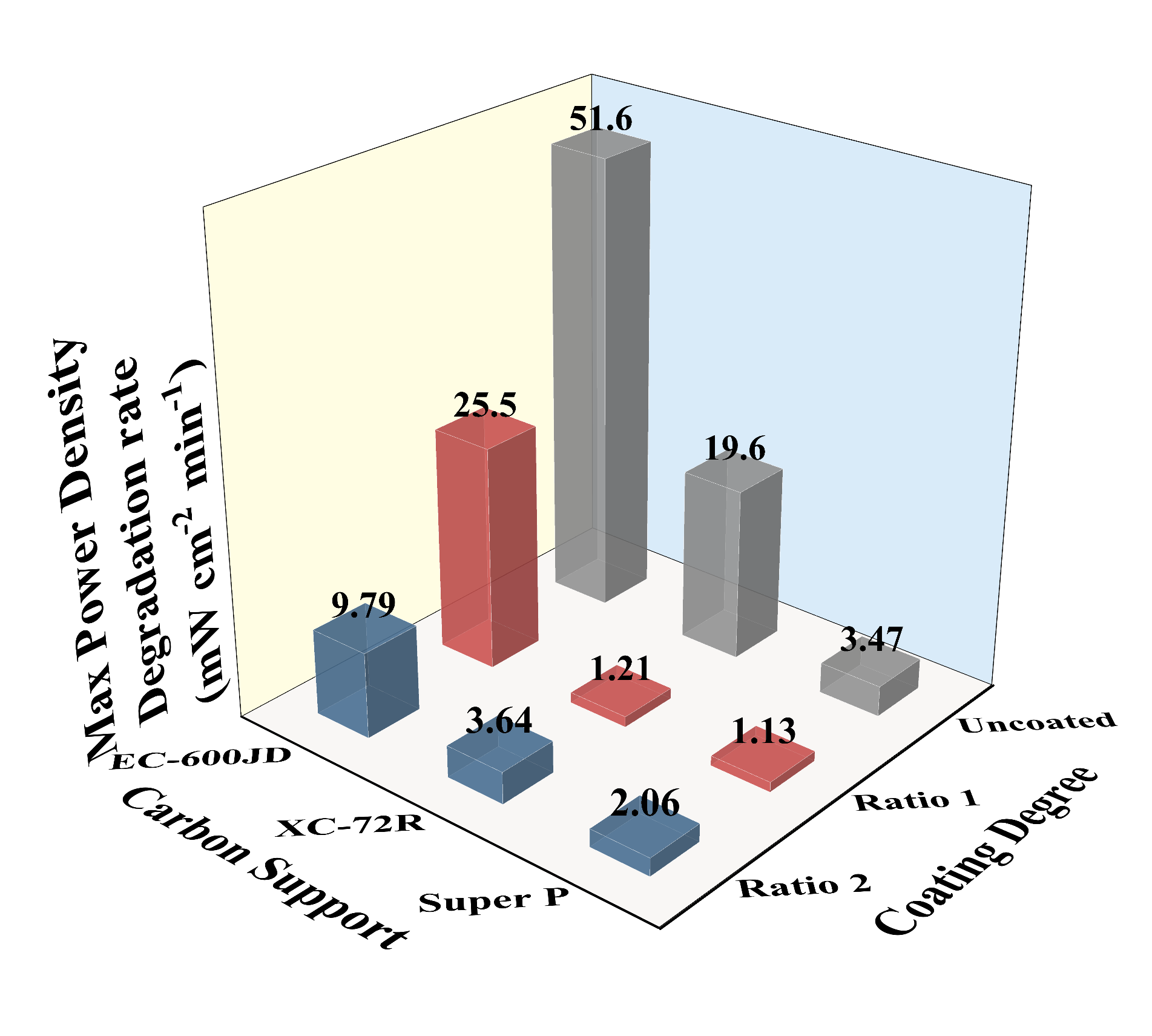


Figure S11. Max power density degradation rates of Pt/C+IrO_2_ RTA and Pt/PANI/C+IrO_2_ RTA under fixed flows (0.5 L/1 L).


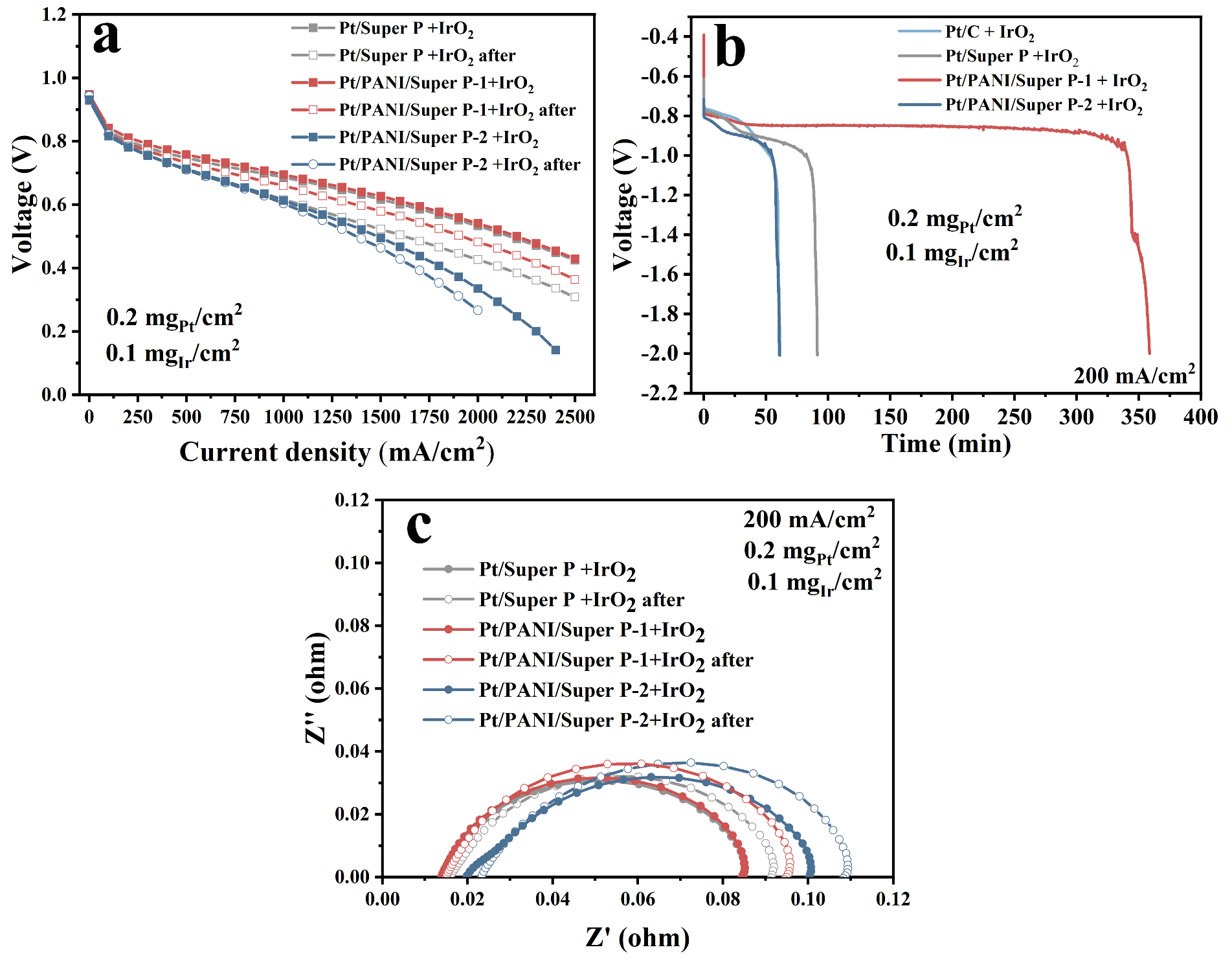


Figure S12. (a) Polarization performance, (b) reversal tests and (c) EIS tests of Pt/Super P +IrO_2_ RTA and Pt/PANI/Super P +IrO_2_ RTAs with 0.2 mg_pt_ cm^-2^ and 0.1 mg_Ir_ cm^-2^ loadings.


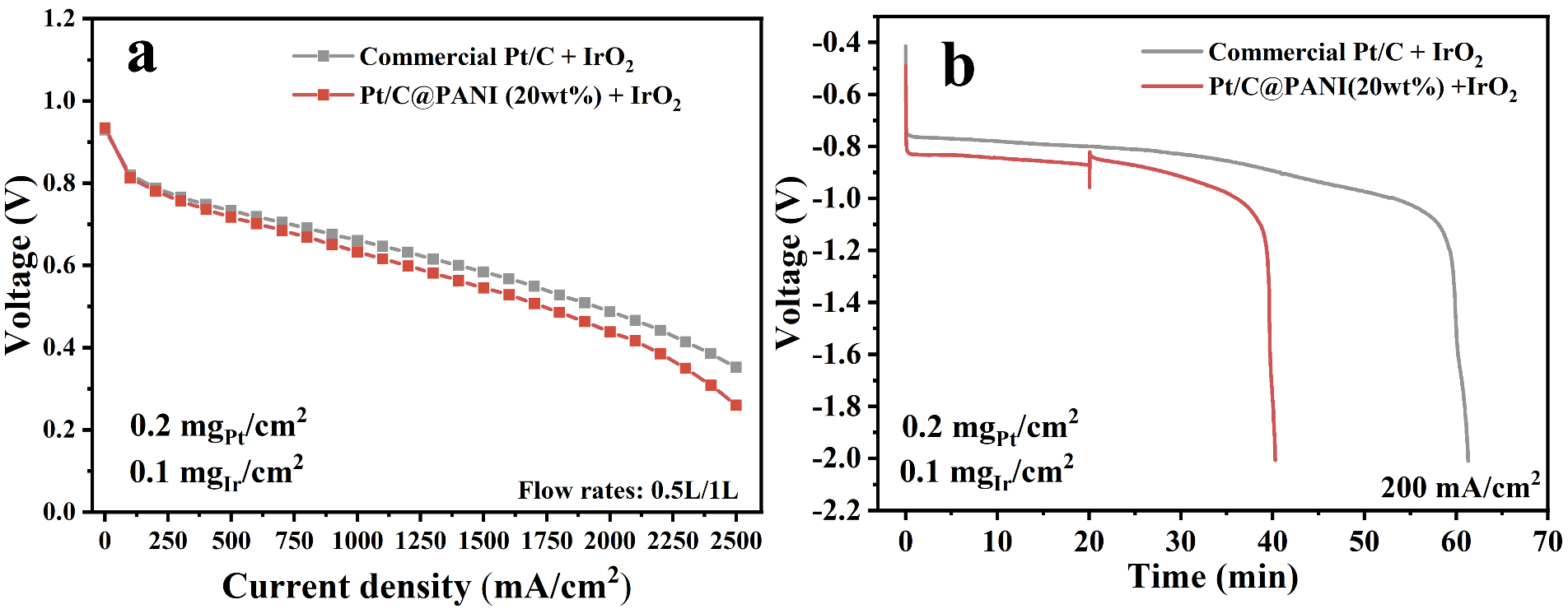


Figure S13. (a) Polarization performance and (b) reversal tests commercial Pt/C and Pt/C@PANI (20wt%) +IrO_2_ RTAs.

**ITC raw data**

As the molecular weight or the polydispersity of the ionomer are not precisely known, the monomer molar concentration is calculated from the mass concentration and equivalent weight. For example, 2.75 mg/mL of Nafion 520 is calculated to be 5.8 mM. The densities of three carbon supports are calculated based on the TGA results. The mole of catalyst binding sites is calculated based on the carbon support density, primary particle size (50 nm diameter), and the projected area of ionomer aggregate (assuming a 3 nm * 10 nm cylinder). For 0.5 mg/mL, the concentration is calculated to be 0.0533 mM. Thermodynamic parameters are extracted from the binding model using this molar basis. The raw data was fitted by a 4-parameter nonlinear fitting model. (Y=a + (X^KD)* (ΔH)/ (X^KD + n^KD)) Specifically, the extracted binding constant is related to the free energy by (|ΔG| = RTln[KA]). Using the enthalpic contribution from the isotherm, entropic contributions are calculated using ΔG = ΔH – TΔS.


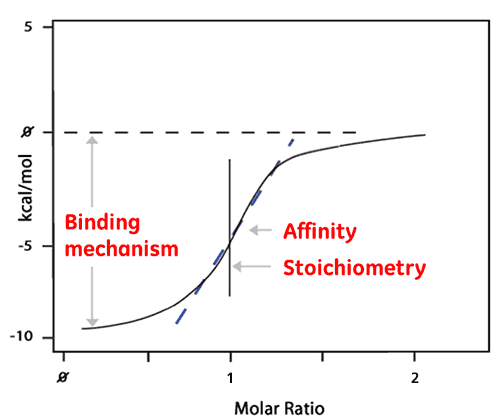


Figure S14. The fitting mechanism of ITC data.


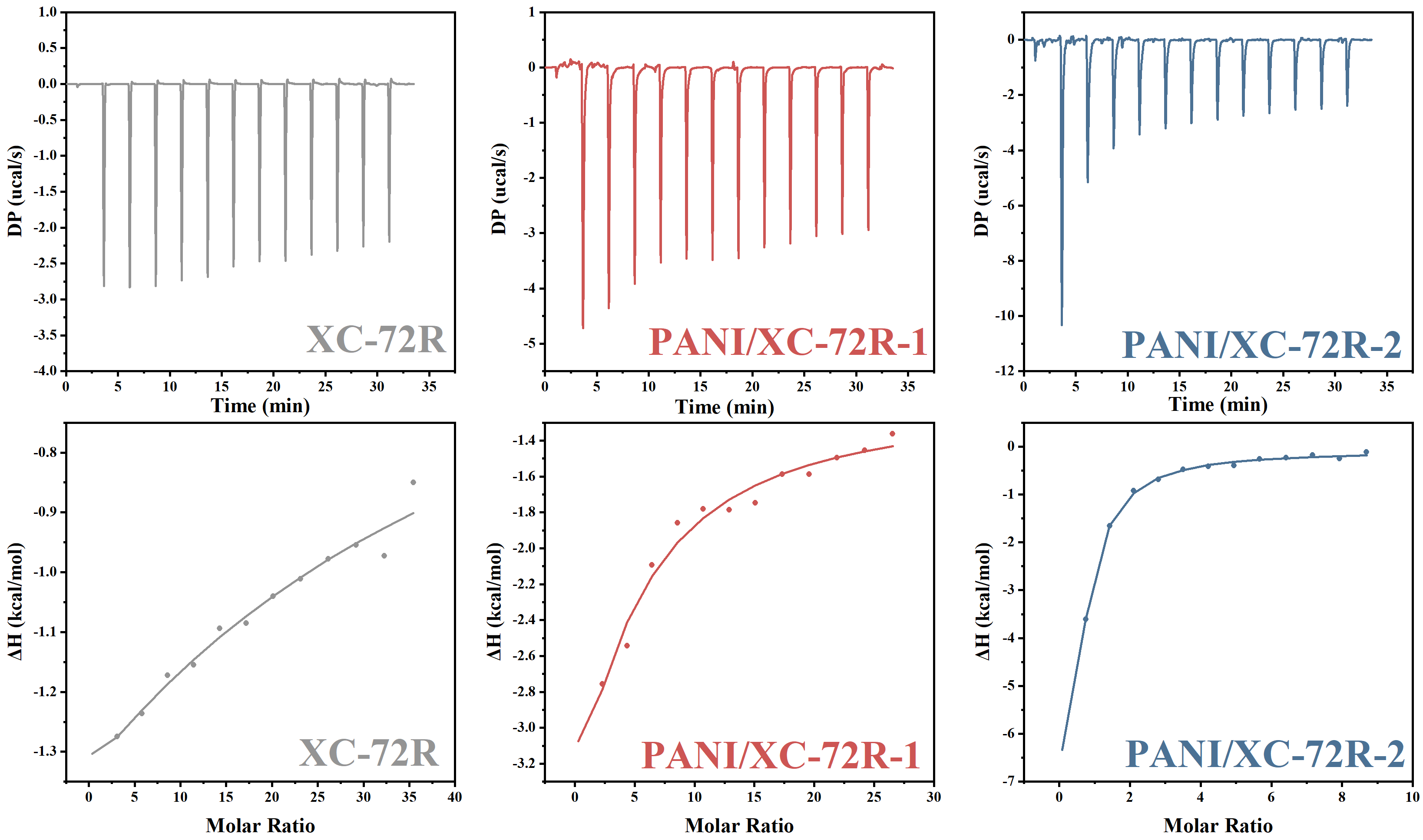


Figure S15. ITC raw data and fitting curves of three carbon supports.


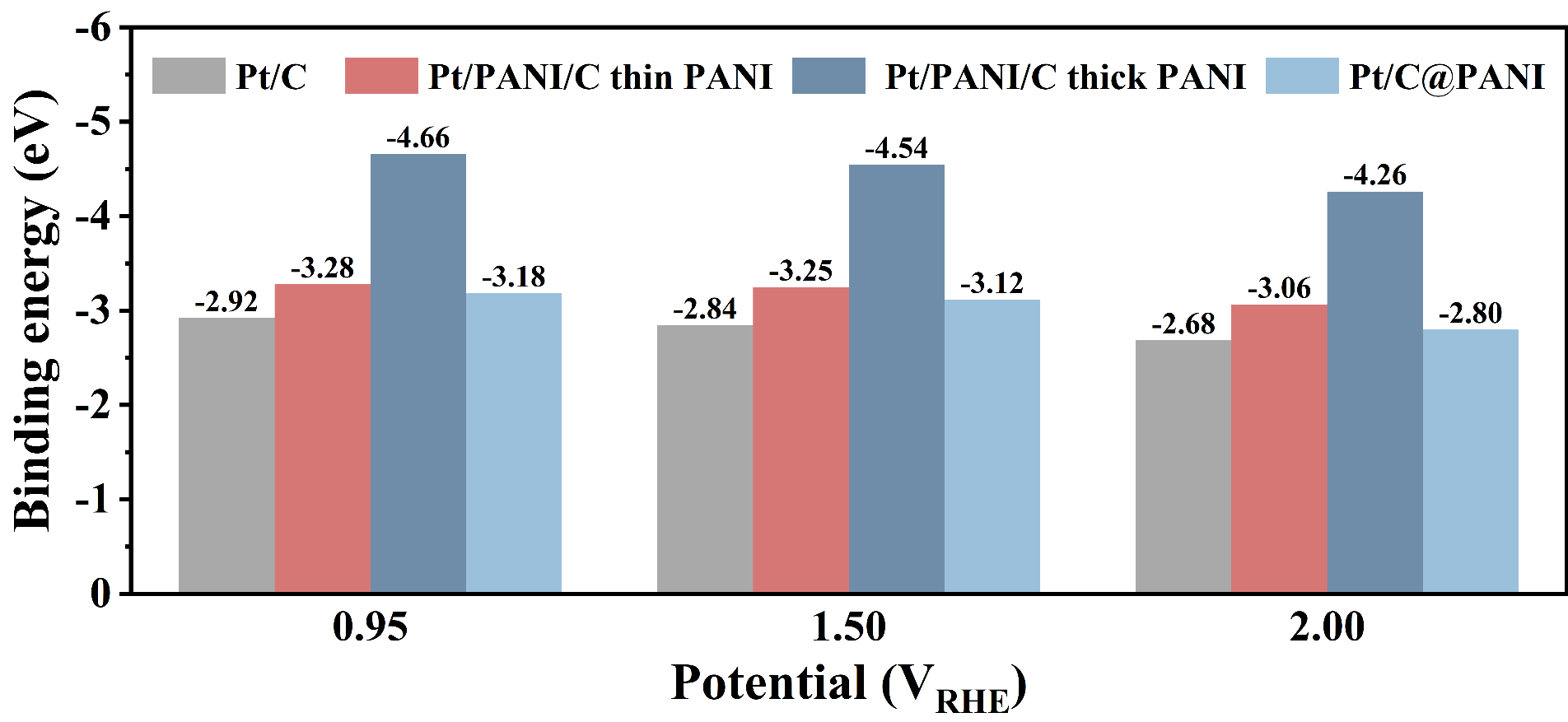


Figure S16. Summary of the binding energy of the calculated structures as a function of three applied potentials (0.95V, 1.50V, and 2.00V).

Table S1. Weight contents obtained by XPS.

| Weight % | Pt 4f | C 1s | O 1s | N 1s |
| --- | --- | --- | --- | --- |
| Pt/Super P | 81.33 | 13.12 | 5.55 | - |
| Pt/PANI/Super P -1 | 81.26 | 6.47 | 10.3 | 1.96 |
| Pt/PANI/Super P -2 | 82.32 | 10.9 | 4.4 | 2.38 |
| Pt/XC-72R | 79.92 | 15.28 | 0.42 | - |
| Pt/PANI/XC-72R -1 | 74.83 | 16.84 | 6.69 | 1.64 |
| Pt/PANI/XC-72R -2 | 79.21 | 5.9 | 13.0 | 1.89 |
| Pt/EC-600JD | 65.00 | 14.27 | 20.73 | - |
| Pt/PANI/EC-600JD -1 | 65.35 | 26.92 | 5.00 | 2.74 |
| Pt/PANI/EC-600JD -2 | 65.08 | 26.94 | 4.76 | 3.22 |

Table S2. Parameters obtained from the impedance fitting results.

|  | R_ohm_ (ohm) | | R_ct,a_ (ohm) | | R_ct,c_ (ohm) | |
| --- | --- | --- | --- | --- | --- | --- |
|  | Before | After | Before | After | Before | After |
| Pt/Super P + IrO_2_ | 0.015496 | 0.020603 | 0.0040169 | 0.0058424 | 0.06423 | 0.069099 |
| Pt/PANI/Super P-1 + IrO_2_ | 0.015699 | 0.022627 | 0.0030874 | 0.0043625 | 0.067688 | 0.069867 |
| Pt/PANI/Super P-2 + IrO_2_ | 0.023128 | 0.025922 | 0.0093569 | 0.016663 | 0.079293 | 0.082476 |
| Pt/XC-72R + IrO_2_ | 0.014245 | 0.020828 | 0.001812 | 0.0026118 | 0.066271 | 0.073718 |
| Pt/PANI/XC-72R-1 + IrO_2_ | 0.014501 | 0.018613 | 0.0024373 | 0.0027346 | 0.064805 | 0.070297 |
| Pt/PANI/XC-72R-2 + IrO_2_ | 0.019095 | 0.020731 | 0.0051255 | 0.0074988 | 0.06986 | 0.071161 |
| Pt/EC-600JD + IrO_2_ | 0.015433 | 0.021371 | 0.003518 | 0.0044242 | 0.066028 | 0.066658 |
| Pt/PANI/EC-600JD -1 + IrO_2_ | 0.015173 | 0.018961 | 0.0042804 | 0.0070305 | 0.060944 | 0.065217 |
| Pt/PANI/EC-600JD -2 + IrO_2_ | 0.01456 | 0.019443 | 0.0032703 | 0.0055814 | 0.063947 | 0.068663 |
